# The Circular RNA Landscape of Non-Small Cell Lung Cancer Cells

**DOI:** 10.3390/cancers12051091

**Published:** 2020-04-28

**Authors:** Nele Van Der Steen, Yanhong Lyu, Anne K. Hitzler, Andrea C. Becker, Jeanette Seiler, Sven Diederichs

**Affiliations:** 1 Division of Cancer Research, Department of Thoracic Surgery, Medical Center―University of Freiburg, Faculty of Medicine, University of Freiburg, 79106 Freiburg, Germany; 2 German Cancer Consortium (DKTK), Partner Site Freiburg, 79106 Freiburg, Germany; 3 Division of RNA Biology & Cancer, German Cancer Research Center (DKFZ), 69120 Heidelberg, Germany

**Keywords:** circular RNA, circRNA, NSCLC, lung cancer, lung adenocarcinoma

## Abstract

The class of circular RNA (circRNA) is characterized by head-to-tail bonds between exons formed by backsplicing. Here, we provide a resource of circRNA expression in a comprehensive panel of 60 lung cancer and non-transformed cell lines (FL3C dataset). RNA sequencing after depletion of ribosomal RNA quantified the expression of circRNA and linear RNA. We detected 148,811 circular RNAs quantified by 2.8 million backsplicing reads originating from 12,251 genes. The number of identified circRNAs was markedly higher using rRNA depletion compared to public polyA-enriched RNA-seq datasets. CircRNAs almost never started in the first exon nor ended in the last exon and started more frequently in earlier exons. Most circRNAs showed high cell line specificity and correlated positively with their linear RNA counterpart. Known cancer genes produced more circRNAs than non-cancer genes. Subsets of circRNAs correlated with cell proliferation, histological subtype or genotype. *CircTNFRSF21* was translated crossing the backsplice site in two different reading frames. Overexpression of *circPVT1*, *circERBB2*, *circHIPK3*, *circCCNB1*, *circSMAD2*, *circTNFRSF21* and *circKIF5B* significantly increased colony formation. In conclusion, our data provide a comprehensive map of circRNA expression in lung cancer cells and global patterns of circRNA production as a useful resource for future research into lung cancer circRNAs.

## 1. Introduction

Circular RNAs (circRNAs) were first described in eukaryotes in the early 1990s and were originally considered to be formed by splicing defects [1,2,3]. Nowadays, numerous circRNAs have been identified by analyzing RNA sequencing data [4]. In contrast to mature messenger RNAs (mRNAs), circRNAs do not possess a polyA tail and a 5′-cap, but are circularized head-to-tail with a 5′−3′ covalent bond [3]. CircRNAs can be identified by mapping RNA sequencing data to a reference genome and identifying sequences that have a reverse orientation [4]. CircRNAs are more stable than their linear counterparts, likely because of protection from exonucleolytic degradation by their covalent head-to-tail bond [5,6].

Backsplicing is dependent on the splicing machinery and uses canonical splice sites [4,6,7,8,9]. Although the details of the process have not been fully elucidated, several rules about the regulation of circRNA formation have been reported. First, a high transcription rate of RNA polymerase II leads to an increase in backsplicing events [10]. Second, there is a competition between circRNA biogenesis and pre-mRNA splicing [8]. Third, backsplicing seems to be facilitated by inverted repeats in the flanking introns [11], e.g., Alu elements [12]. Fourth, RNA binding proteins might promote the looping of RNA through dimerization upon binding of their respective motifs, e.g., Quaking (QKI) [13] and FUS [14] or reduce looping by reducing complementarity, e.g., ADAR1 [11]. In general, cellular proliferation leads to a downregulation in circRNA expression [15], although a small subset of circRNAs shows an increase upon proliferation [16]. While many circRNAs contain in silico predicted open reading frames (ORFs) with internal ribosomal entry sites (IRES), only a small number of these proteins/peptides have been detected or experimentally verified [17,18,19,20,21,22].

Currently, the majority of studies linking circRNAs to cancer focus on their function as miRNA sponges [23,24], thus influencing oncogenic pathways [25]. Additionally, peptides arising from circRNAs can function as decoys and thus influence cancer growth, e.g., the peptide translated from *circβ-catenin* protects full-length β-catenin from phosphorylation by GSK3β and subsequent degradation [26]. Finally, circRNAs can influence cell proliferation by protein scaffolding, e.g., the *circFOXO3* RNA forms a complex with CDK2 and p21 to prevent cell cycle entry [27].

Lung cancer, representing 11.8% of all cancer diagnoses, is the most commonly diagnosed cancer type worldwide [28]. It is also the leading cause of cancer-related deaths worldwide, with 1.8 million deaths per year, which represents 18.4% of all cancer-related deaths [28]. The most common type of lung cancer is non-small cell lung cancer (NSCLC), representing 85% of lung cancers. NSCLC can be further divided into adenocarcinoma (LUAD) and squamous cell carcinoma (LUSC) subtypes [29]. While many pathways have been linked to lung tumorigenesis like EGFR or KRAS [30], the underlying mechanisms remain unknown in many cases with non-coding RNAs emerging as additional players in carcinogenesis and tumor progression like *MALAT1* [31], *PVT1* [32] or *linc00673* [33].

Due to their high stability, circRNAs are considered as good candidates for new biomarkers [34]. A specific example for lung cancer are the circRNAs that originate from the EML4-ALK fusion gene, F-circEA, which can be detected in plasma samples of these patients [35,36]. Moreover, circRNAs might serve as good predictive biomarkers for response to therapy [37,38,39].

Here, we describe the circRNA landscape in non-small cell lung cancer cell lines. After assembling a platform of 60 lung cell lines (57 lung cancer cell lines and 3 non-transformed lung cell lines), we used deep sequencing of rRNA-depleted RNA for profiling the exonic circRNAs and the linear RNA transcriptome. We describe the general characteristics of this dataset taking into account differences between the gene level (all circRNAs of one gene were grouped during analysis) and the backsplice level (all circRNAs were considered separately during analysis). Furthermore, we link circRNAs to specific phenotypes and genotypes in non-small cell lung cancer.

## 2. Results

### 2.1. circRNA Detection in Lung Cancer Cells after rRNA Depletion

We assembled a lung cell line panel of 60 lung cell lines, consisting of 50 adenocarcinoma cell lines, seven other NSCLC cell lines and three non-transformed cell lines (Supplementary Table S1), which we named the Freiburg Lung Cancer Cell Collection (FL3C). After total RNA isolation, the rRNA was depleted and RNA of all cell lines was sequenced in replicate (*n* = 175 with two or three replicates per cell line) and mapped to a reference genome to generate the linear RNA dataset. Next, we identified circRNAs by identifying reverse mapped reads resulting from backsplicing and constructed a separate circRNA dataset. In total, we found 2.8 million backsplicing reads compared to 3.8 billion reads mapping linearly to the genome.

Overall, we found on average 731 circRNA reads per million reads in our dataset based on rRNA depletion prior to RNA sequencing. At the gene level, we detected circRNAs for 12,251 genes and provide the full dataset for 60 cell lines in Supplementary Table S2. At the backsplice level, we identified 148,811 individual circRNAs and provide the full dataset in Supplementary Table S3.

**Figure 1:**
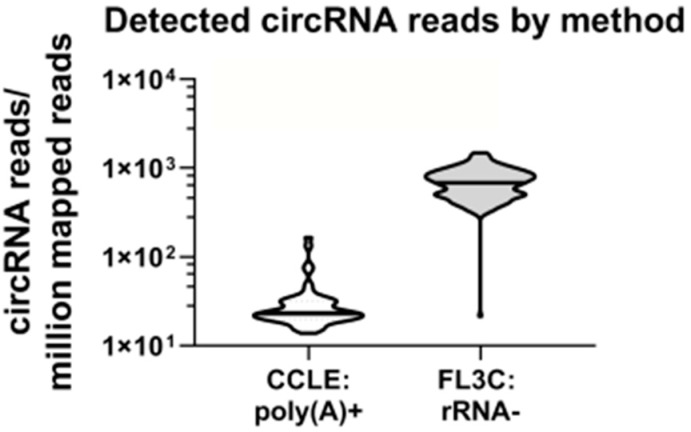
Detected circRNA reads by method. This violin plot compares the detected circRNA reads per million mapped reads in the CCLE and the FL3C database.

We compared our dataset to a publically available dataset of the Cancer Cell Line Encyclopedia (CCLE) [40,41] from which we retrieved RNA sequencing data after polyA-enrichment from 54 cell lines (single replicate) overlapping with our panel. Notably, these data contained 25-fold less circRNA reads (Figure 1).

Next, we looked at the enrichment in polyA stretches between the CCLE and the FL3C datasets. In the CCLE dataset, 11,441 circRNAs were identified, of which 5587 were overlapping with the FL3C dataset, which contained in total 148,811 circRNAs. When we compared the top 100 most strongly expressed circRNAs, 15 showed no overlap and 85 were shared between the datasets. Of the shared circRNAs, 69% contained polyA stretches of 5 or more consecutive As, versus only 33% of the circRNAs that were uniquely identified in the FL3C dataset. In conclusion, there may be a trend towards enrichment in polyA stretches in circRNAs that were identified by sequencing after polyA selection. In brief, efficient circRNA detection was feasible after rRNA depletion and subsequent RNA sequencing while the circRNA content detected in polyA-enriched RNA was significantly lower.

### 2.2. Lung Cell Line Transcriptomics and the circRNA Landscape

**Figure 2:**
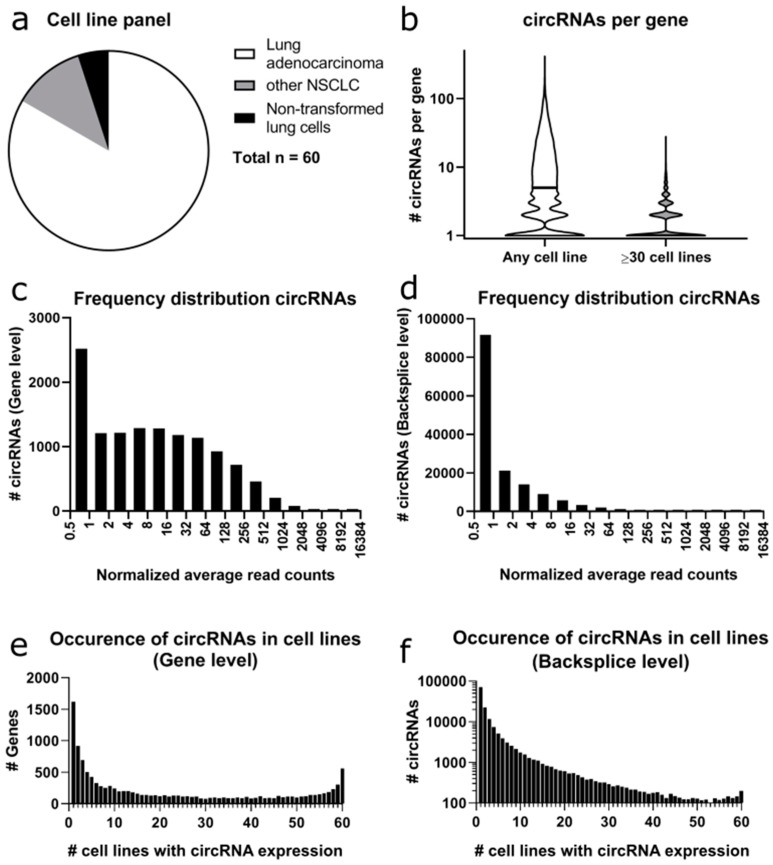
General dataset characteristics. (**a**) The FL3C cell line panel consists of 60 human lung cell lines (50 cell lines have been described as adenocarcinoma, 7 cell lines as non-adenocarcinoma NSCLC and 3 cell lines as non-transformed lung cells). (**b**) circRNAs per gene. The violin plot depicts the number of circRNAs (different backsplice sites) per gene in the FL3C dataset before and after selection of the circRNAs that are expressed in at least half of the cell lines. The black line depicts the median. (**c**) Frequency distribution of circRNAs at the gene level. Bar graph depicting the distribution of the sum of the circRNA reads found in one gene in the FL3C dataset, i.e., how many genes (y-axis) show a total number of circRNA reads indicated on the x-axis. (**d**) Frequency distribution of circRNAs at the backsplice level (as in (**c**)). (**e**) Occurrence of circRNAs in cell lines at the gene level. The graph depicts the distribution of genes according to the number of cell lines with occurrence of a circRNA for this gene. (**f**) Occurrence of circRNAs in cell lines at the backsplice level. The graph depicts the distribution of circRNAs according to the number of cell lines with occurrence of the circRNA.

After quantifying the linear and circular RNA read counts in the FL3C dataset (Figure 2a), we calculated the number of different backsplicing sites per gene representing different circRNAs derived from the same gene (Figure 2b). For the entire dataset, we found on average 12 and at median five different backsplice sites per gene, with a minimum of one (in 2506 out of the 12,251 genes with circRNAs detected) and a maximum of 418 for *BIRC6*. Next, we repeated the analysis only for the circRNAs that were expressed in at least 30 out of the 60 cell lines focusing on more broadly detectable circRNAs. This filter resulted in 4124 circRNAs at the gene level and 5232 circRNAs at the backsplice level. In this subset, we found on average two and at median only one circRNA per gene with *PTK2* expressing the maximum number of circRNAs with 28 unique backsplice sites. For 1170 genes, more than one circRNA was expressed in the majority of cell lines.

The normalized circular read counts (average of 2–3 replicates, normalized to the library size) at the gene level ranged from 14,487 to 0.17, with an average of 70.51 circular reads at gene level in all cell lines (Figure 2c). The genes with the highest circRNA read counts were *CYP24A1* (14,487) and *ASPH* (13,783).

This result is mirrored at the backsplice level (Figure 2d), with the circRNA *circCYP24A1* with a backsplice site linking exons 11 to 3 having 14417 counts and circRNA *circASPH* (backsplice site: exon 3-2) having 10,493 counts. Overall, the normalized counts at the backsplice level ranged from 14,417 to 0.17, with an average of 5.8 reads per unique circRNA.

When comparing the expression of circRNAs across all cell lines tested, there were major differences between the analysis at the gene level (Figure 2e) and the backsplice level (Figure 2f). At the gene level, 13.2% of genes expressed circRNAs only in a single cell line, while at the backsplice level, 47.3% of the circRNAs were only expressed in a single cell line. At the gene level, 14.1% of the genes expressed circRNAs in ≥90% of the cell lines, while at the backsplice level, only 0.7% of the circRNAs were expressed in ≥90% of cell lines.

For six selected circRNAs, we verified their expression in lung cancer cell lines using RT-PCR in comparison to the total RNA of the respective gene (Supplementary Figure S1a). To confirm the circularization of the detected amplicons, we treated the RNA with the exonuclease RNase R. The ratio of circular to total RNA markedly differed between the genes with *PVT1* having the largest circular fraction of its total RNA and *CCNB1* having the lowest circular fraction of its total RNA (Supplementary Figure S1b).

### 2.3. The First and Last Exon Are Depleted From circRNAs

**Figure 3:**
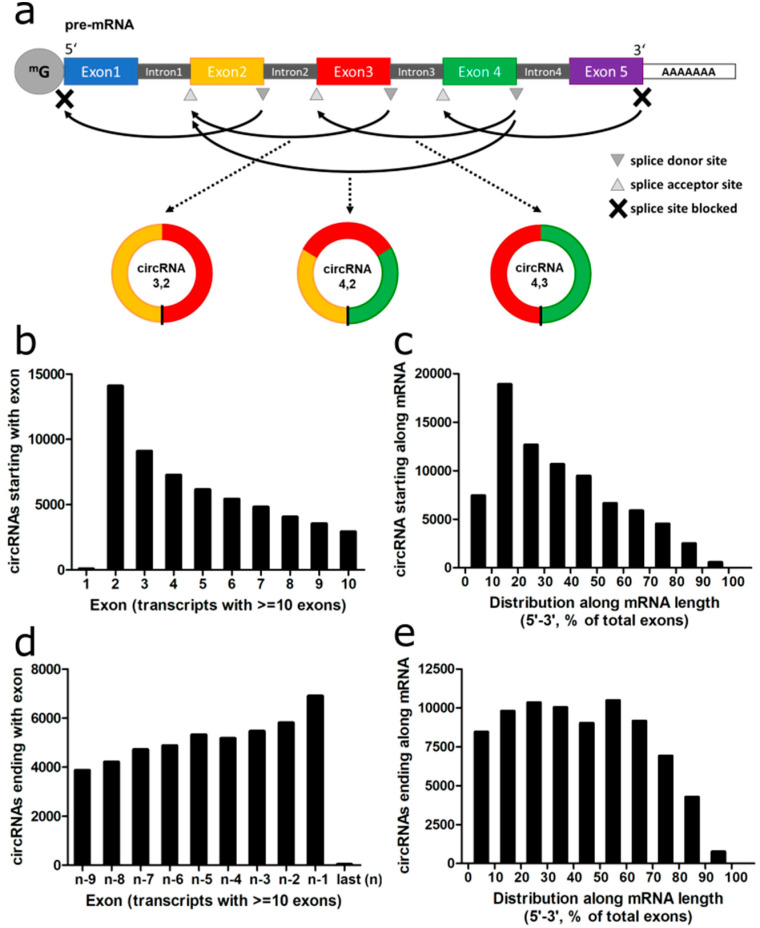
Prevalence of exons in circRNAs. (**a**) Schematic depiction of alternative backsplicing events. (**b**) Number of detected circRNAs that use an exon as acceptor exon. (**c**) Number of detected circRNAs that use a percentile of mRNA as acceptor exon. (**d**) Number of detected circRNAs that use an exon as donor exon. (**e**) Number of detected circRNAs that use a percentile of mRNA as donor exon.

We then analyzed the representation of the exons along the mRNA within circRNAs. First, we analyzed the acceptor exon of the circRNAs, i.e., the “start” exon appearing first in the linear RNA to which a later exon, the donor exon, was backspliced (Figure 3a).

The second exon of the transcript was more frequently serving as acceptor exon than the first exon with a 78-fold difference for all circRNAs and a 126-fold difference for circRNAs detected with at least two reads and derived from transcripts with at least 10 exons (Figure 3b). When looking along the entire mRNA, the first 10% of exons were 2.5-fold less often serving as acceptor exons in comparison to the second 10%. The second exon was the most prevalent acceptor exon for circularization, while this steadily decreased over the subsequent exons (Figure 3c).

We repeated the analysis for the donor exon of the circRNA. The last exon of the transcript was less often serving as donor exon in comparison to the second to last exon with a 62-fold difference across all circRNAs and a 1778-fold difference for transcripts of ≥10 exons and at least two circRNA reads (Figure 3d). The last 10% of the exons were 1.4-fold less often donor exons in comparison to the second to last increment (Figure 3e). While the rate of circRNAs using an exon as donor was declining with decreasing exon number similar to the acceptor exons along the mRNA, this effect was much less pronounced than for the acceptor exons. In summary, the first and last exons very rarely were included in circRNAs, while the second exon was most frequently serving as acceptor exon.

### 2.4. circRNAs Separate Lung Cancer Cells from Non-Transformed Lung Cells

**Figure 4:**
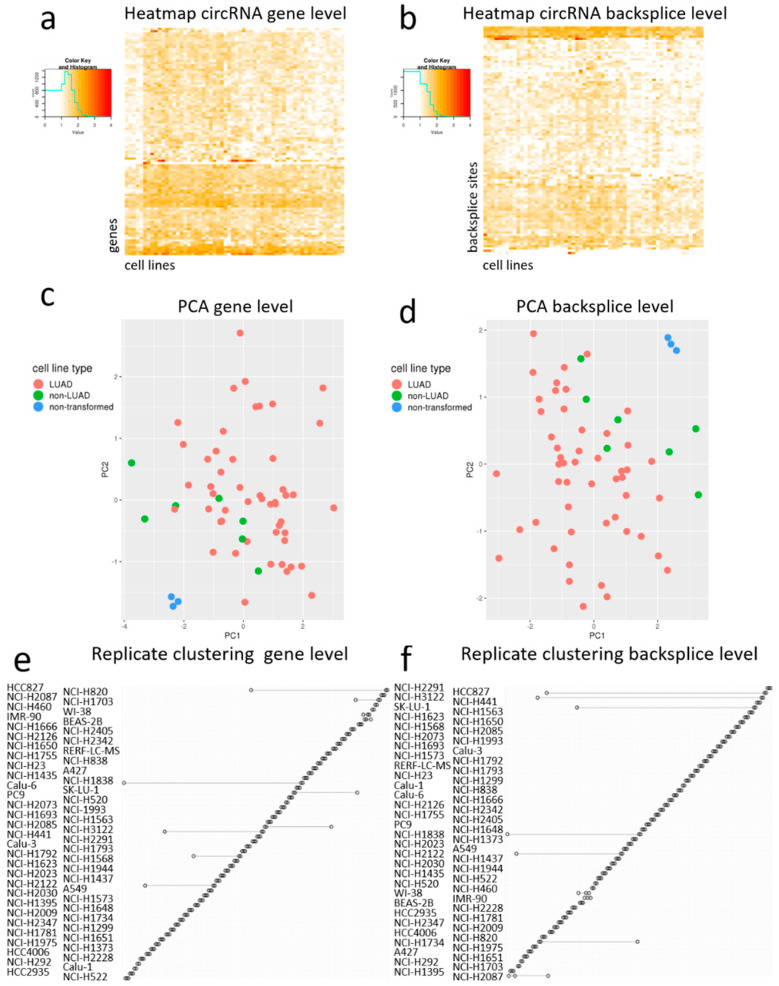
Clustering and reproducibility of circRNA expression at the gene or backsplice level. (**a**) Heatmap illustrating clustering of the cell lines by the top 100 highest expressed circRNAs grouped per gene. (**b**) Heatmap illustrating clustering of the cell lines by the top 100 highest expressed unique circRNAs. (**c**) Principal component analysis (PCA) of the cell lines based on the top 100 highest expressed circRNAs grouped per gene. (**d**) Principal component analysis of the cell lines based on the top 100 highest expressed unique circRNAs. (**e**) Clustering of the cell line replicates at gene level. (**f**) Clustering of the cell line replicates at backsplice level.

Next, we constructed heatmaps, based on the top 100 genes producing most circRNAs (Figure 4a) and on the top 100 most highly expressed circRNAs (Figure 4b). Both heatmaps (gene level and backsplice level) showed the high abundance of circRNAs derived from *CYP24A1* and *ASPH*. Moreover, the heatmap for individual circRNAs also showed a high expression, e.g., of *circERBB2* specifically in the Calu-3 cell line, which contained an *ERBB2* amplification, thus showing a link with the genotype of the cell line. When performing unsupervised clustering for the cell lines, the non-transformed cell lines clustered together, both at the gene and the backsplice level. The principal component analysis also depicted the non-transformed cell lines clustering separately from the adenocarcinoma and non-adenocarcinoma NSCLC cell lines (Figure 4c,d). To a lesser extent, the non-adenocarcinoma cell lines also clustered together at both levels. The replicate clustering showed a very good clustering reflecting high reproducibility for the large majority of the cell line replicates both at the gene and the backsplice level (Figure 4e,f).

### 2.5. Positive Correlation between Circular and Linear RNA Expression

**Figure 5:**
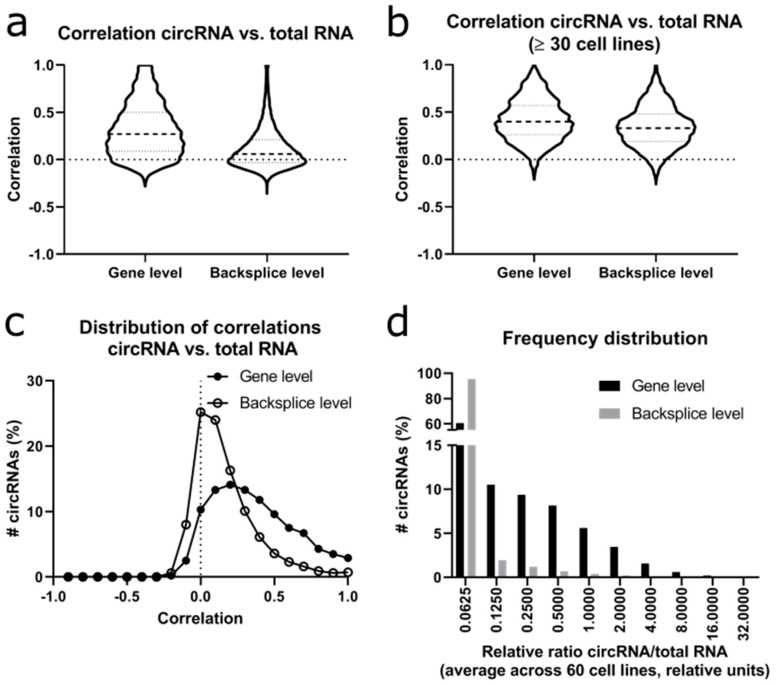
Correlation between circRNA and total (linear) RNA. (**a**) Distribution of correlation coefficients between circRNA and total RNA (detected by analysis of linear reads, which may, outside of the backsplice region, also be derived from circRNAs) at gene level (circRNA grouped per gene) and backsplice level (unique circRNAs) for the whole dataset. Dashed lines depict the median, dotted lines depict the first and third quartiles. (**b**) Distribution of correlation between circRNA and total RNA at gene and backsplice level of RNAs that are expressed in ≥30 cell lines. Dashed line depicts the median, dotted lines depict the first and third quartiles. (**c**) Distribution of correlation between circRNA and total RNA at gene and backsplice level. (**d**) Relative circRNA/total RNA ratio, both at gene and backsplice level (rel. units).

Since circRNAs are formed from the linear transcript, we calculated the correlation distribution between circRNA and “linear” RNA derived from the same gene. We refer to the signals derived from the linear RNA analysis as “total RNA” since all reads of the circRNA outside of the backsplice region would be indistinguishable from linear reads and would thus be included into this analysis of linear RNAs. When considering the total dataset, there was a positive correlation at gene level with a median correlation of 0.27 (Figure 5a), whereas this correlation was much lower at the backsplice level when considering each of the circRNAs separately with a median correlation of only 0.06. When repeating this analysis for circRNAs that were expressed in ≥30 cell lines, the difference between gene level and backsplice level decreased (Figure 5b), with a median correlation of 0.40 at the gene level and of 0.27 at the backsplice level. So, the majority of the well expressed circRNAs showed a positive correlation to the underlying total (linear) RNA, while only very few examples showed any negative correlation. At the gene level, 86.9% of circRNAs showed a positive correlation with their co-derived total (linear) RNA, versus 66.2% of circRNAs at backsplice level (Figure 5c). Next, we calculated the relative ratio of circRNA/total RNA (Figure 5d).

Importantly, the calculated ratios of circRNA/total RNA did not reflect an absolute ratio of molecules in the cell but a relative number dividing the number of circRNA reads (only backsplice reads, normalized only for library size) to the FPKM of the linear reads (all reads along the transcript, normalized for library size and gene length). When considering the average ratio of circRNA/total RNA over the 60 cell lines, the median ratio was 0.03 at the gene level versus <0.01 at the backsplice level.

Overall, the circRNA/total RNA ratios showed a broad distribution with a range from <0.01 to >23 for *MUC16* indicating that circRNAs can have largely varying ratios to the total RNAs derived from the same locus. These values may be used to select highly efficiently circularized transcripts.

When comparing the ratios in individual cell lines, the highest ratio of 561.3 at gene level was identified for the *EFCAB12* (EF-Hand Calcium Binding Domain 12) gene, but this circRNA was only expressed in 5/60 cell lines. The second highest ratio of 427.8 was reached by the *OTUD7A* (OTU Deubiquitinase 7A) gene, which was expressed in 10/60 cell lines. When a cut-off of expression in ≥30 cell lines was used, *MUC16* showed the highest ratio of 248.8 with an expression in 40 cell lines. Focusing on individual circRNAs at the backsplice level, *OTUD7A* (backsplice site: 4–3) had the highest circRNA/total RNA ratio of 427.8 but was only expressed in three cell lines. *MUC16* (backsplice site: 69–68) was in the top 10 with a ratio of 248.8 and was expressed in 27 cell lines.

In summary, circRNAs showed a positive correlation with the total linear RNA derived from the same gene, but the ratios varied largely when comparing individual cell lines, genes or circRNAs.

### 2.6. Cancer Genes Produce More Circular RNAs

**Figure 6:**
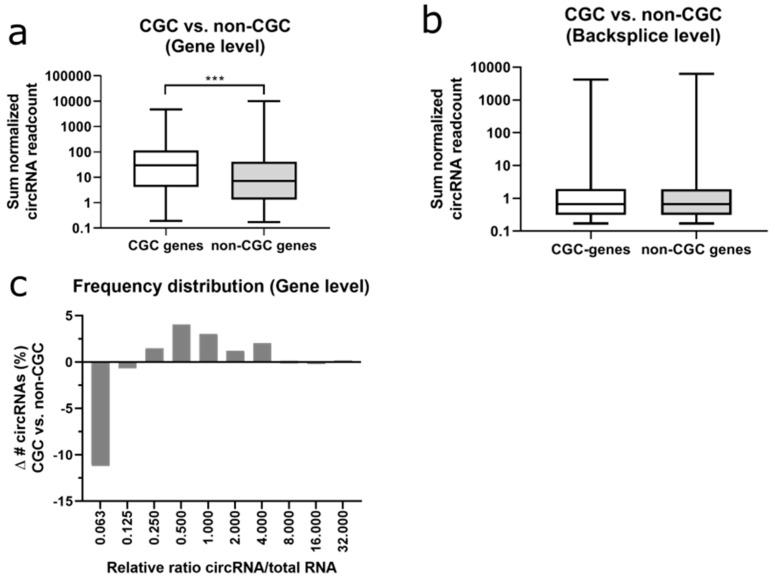
CircRNAs in Cancer Genes (CGC, v90). (**a**) Sum of normalized circRNA read counts in Cancer Gene Census (CGC) genes vs. non-CGC genes at the gene level. *T*-test: *p*-value < 0.001 (***). (**b**) idem at backsplice level. (**c**) Frequency distribution of the difference in number of circRNAs between CGC and non-CGC genes for the relative circRNA/total RNA ratios.

To assess the circRNA subset derived from known cancer genes, we divided the FL3C dataset into cancer genes and non-cancer genes according to the Cancer Gene Census (CGC) of COSMIC (version 90) [42]. At the gene level, 4.6% of circRNAs originated from CGC-genes (Figure 6a), while CGC genes represent only 2.8% of all coding genes. Average normalized read counts were significantly higher for cancer genes (CGC) as compared to non-CGC-listed genes (2.1-fold, *T*-test *p* = 1.8 × 10^−5^). At the backsplice level, even 8.4% of circRNAs originated from cancer genes (Figure 6b). In contrast to the gene level, there was only a statistically insignificant 1.1-fold change in average normalized read counts between both categories at the backsplice level.

CircRNAs with a low circRNA/total RNA ratio were less frequently originating from cancer genes (Figure 6c). In summary, cancer genes were generating significantly more circRNAs as compared to non-CGC-listed genes, but this difference was not found at the individual circRNA level.

### 2.7. A Subset of Circular RNAs Correlates with Proliferation

**Figure 7:**
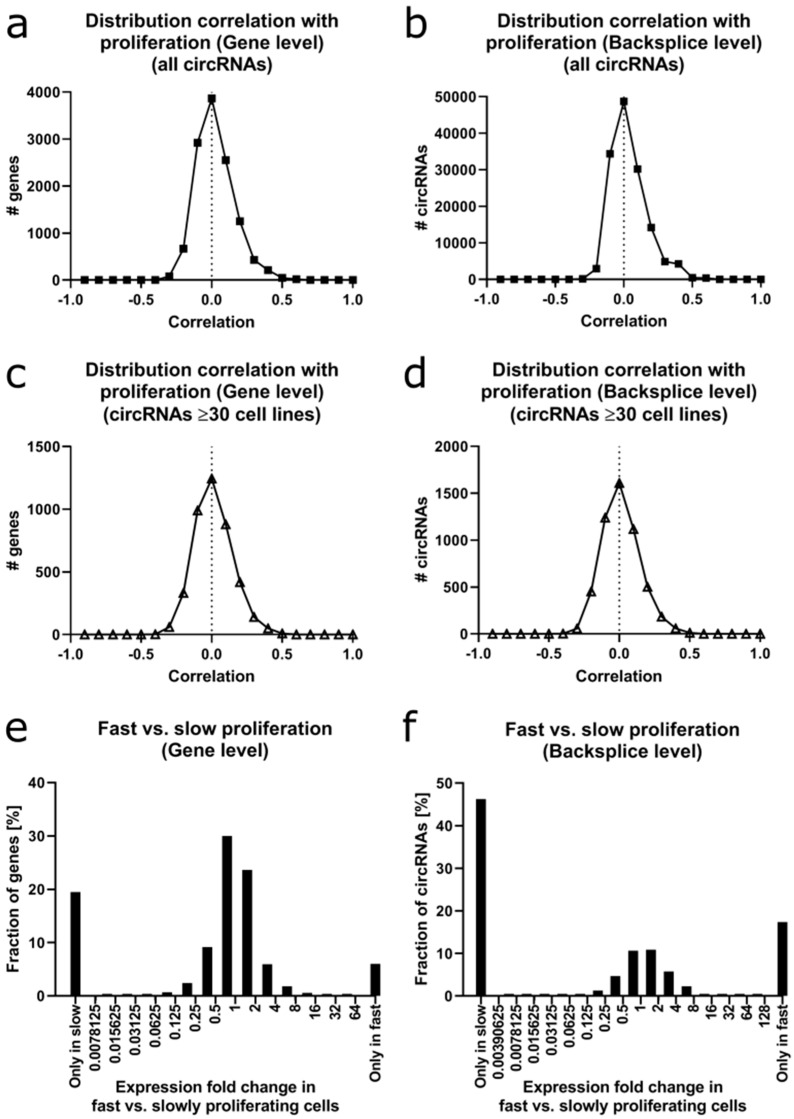
Correlation of circRNAs with cell proliferation. (**a**) Distribution of the correlation of circRNAs with proliferation rate at gene level (circRNAs grouped per gene) across the whole dataset. (**b**) Distribution of the correlation of circRNAs with proliferation rate at backsplice level (unique circRNAs) across the whole dataset. (**c**) Same as (**a**) with a cut-off of circRNA detection in ≥30 cell lines. (**d**) Same as (**b**) with a cut-off of circRNA detection in ≥ 30 cell lines. (**e**) Distribution of the fold change of circRNAs between fast and slow proliferating cell lines at the gene level. (**f**) Same as (**e**) at the backsplice level.

We determined the correlation of circRNAs with the proliferation rate of the FL3C cell lines, which we had experimentally determined before (see Supplementary Table S1). At the gene level, the correlation coefficient ranged from +0.58 to −0.47 for the whole dataset (Figure 7a).

For circRNAs expressed in ≥30 cell lines, the correlation ranged from +0.52 to −0.45 (Figure 7c). The majority of the circRNAs showed as expected little to no correlation with the proliferation rate.

At the backsplice level, the correlation ranged from +0.65 to −0.45 for the whole dataset (Figure 7b), and +0.49 to −0.45 for circRNAs expressed in ≥30 cell lines (Figure 7d).

As an orthogonal analysis to the correlation analysis, we divided the cell lines into two groups based on their proliferation rate. Cell lines with ≥5-fold increase in cell count within 72 h were considered to be fast proliferating (11 cell lines). Cell lines with ≤3-fold cell count increase within 72 h were considered as slowly proliferating (21 cell lines). At the gene level (Figure 7e), circRNAs from 17.8% of the 12,251 genes were only expressed in slowly proliferating cell lines versus 5.5% only expressed in fast proliferating cells. At the backsplice level (Figure 7f), 33.8% of the unique circRNAs were only expressed in slowly proliferating cells versus 12.7% of the circRNAs only expressed in fast proliferating cells. Focusing on the fold change of expression between fast and slowly proliferating cells, 49.0% of the circRNAs had a fold change between 0.5 and 2.0 when considering the whole dataset versus 15.7% at the backsplice level. These percentages rose to 88.5% and 84.5%, respectively, when taking only the circRNAs in consideration that were expressed in ≥30 cell lines.

Next, we selected circRNAs derived from CGC genes which were expressed in ≥30 cell lines and showed a strong positive and/or negative correlation between circRNA expression and proliferation.

At the gene level, three genes drew our attention: *SMAD2*, *MET* and *DEK*. *SMAD2* and *MET* showed a positive correlation between proliferation and circRNA expression (+0.38 and +0.32, respectively) and between proliferation and total RNA expression (+0.44 and +0.23, respectively). In contrast, *DEK* showed a negative correlation between its circRNA expression and proliferation (−0.34), while there was notably no correlation between its total (linear) RNA expression and proliferation (−0.01).

At the backsplice level, two isoforms of *circSMAD2* were identified with a high correlation to proliferation: backsplice site exon 6–exon 2 (+0.37) and exon 6–exon 3 (+0.35). *MET*, (backsplice site exon 2–exon 2) showed a correlation of +0.32 with proliferation. For *DEK*, also two circRNAs were identified with a large negative correlation to proliferation: backsplice site exon 9–exon 8 (−0.32) and exon 9–exon 3 (−0.31). For the *KIF5B* gene, two different circRNAs showed opposite correlations to proliferation: backsplice site exon 24–exon 20 (+0.29) and backsplice site exon 20–exon 18 (−0.29), while the total RNA showed a weaker negative correlation to proliferation of −0.16.

**Figure 8:**
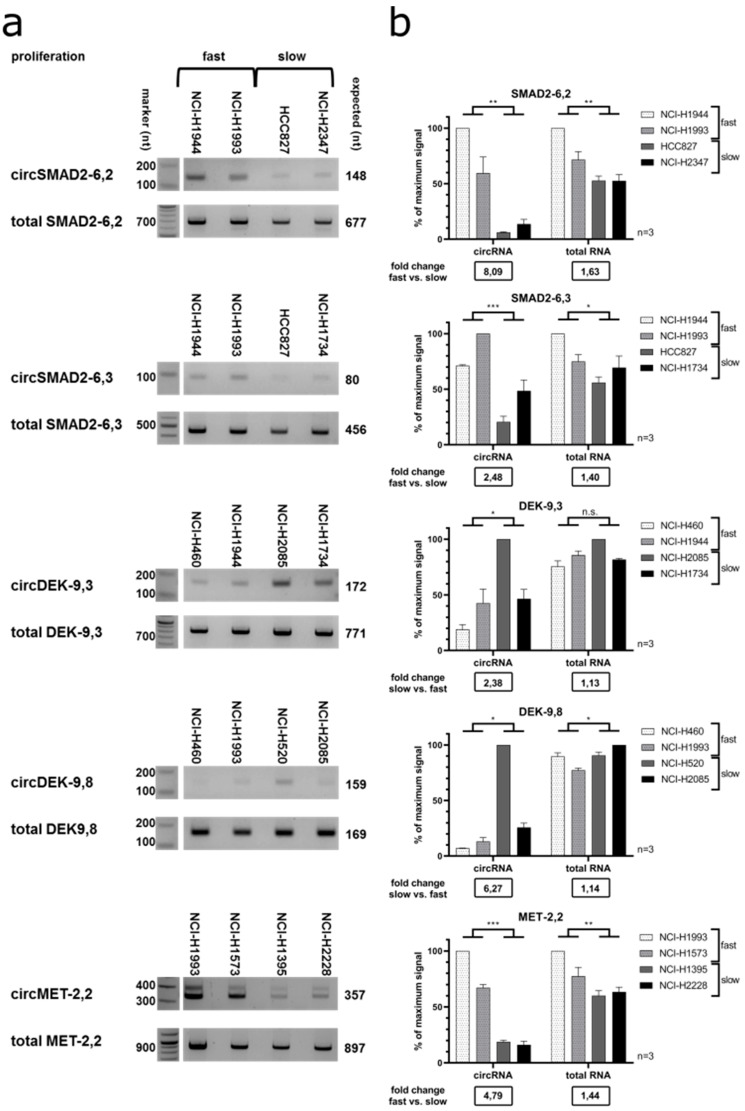
Validation of proliferation-correlated circRNAs in fast vs. slowly proliferating cell lines. (**a**) Representative images after gel electrophoresis. Per row the first two cell lines were fast proliferating (≥5-fold increase in cell count in 72 h), the last two cell lines were slowly proliferating (≤3-fold cell count increase in 72 h). Numbers depict marker size (left) and expected fragment sizes (right). (**b**) Signal strength of the bands after gel electrophoresis was quantified (*n* = 3). The relative signal (% of highest signal in each group) is depicted for circRNA and total RNA, respectively. *T*-test: not significant (n.s.), *p*-value < 0.05 (*), *p*-value < 0.01 (**), *p*-value < 0.001 (***). Below the graph, the fold change of average relative signal between the groups is shown.

We validated these results with semi-quantitative RT-PCR in a subset of cell lines with either fast or slow proliferation (Figure 8). *CircSMAD2* (backsplice site exon 6–exon 2) was upregulated in fast proliferating cell lines and showed a significant 8.1-fold change, versus a significant 1.6-fold change for the total *SMAD2* RNA. The stronger differential expression of *circSMAD2-6,2* than the total *SMAD2* mRNA was also verified by RT-qPCR (Supplementary Figure S2). This difference was less pronounced for *circSMAD2* (backsplice site exon 6–exon 3) resulting in a 2.5-fold change versus a 1.4-fold for the total RNA. For the *circDEK* isoforms, backsplice site exon 9–exon 8 was most strongly downregulated in fast proliferating cell lines, with a significant 6.3-fold change versus a significant only 1.1-fold change for the total RNA. *CircDEK* backsplice site exon 9–exon 3 had a significant 2.4-fold change versus a non-significant 1.1-fold change for the total RNA. *CircMET* showed a significantly higher expression in fast proliferating cell lines with a 4.8-fold change versus a significant only 1.4-fold change for the total RNA (Figure 8a,b).

Overall, only a small subset of circRNAs correlated positively with cell proliferation or was differentially expressed between fast and slowly proliferating cells. Interestingly, circRNAs and linear RNAs or different circRNAs originating from the same gene may demonstrate differences in correlation with cell proliferation.

### 2.8. Circular RNAs Correlate with Histological or Genetic Classification

**Figure 9:**
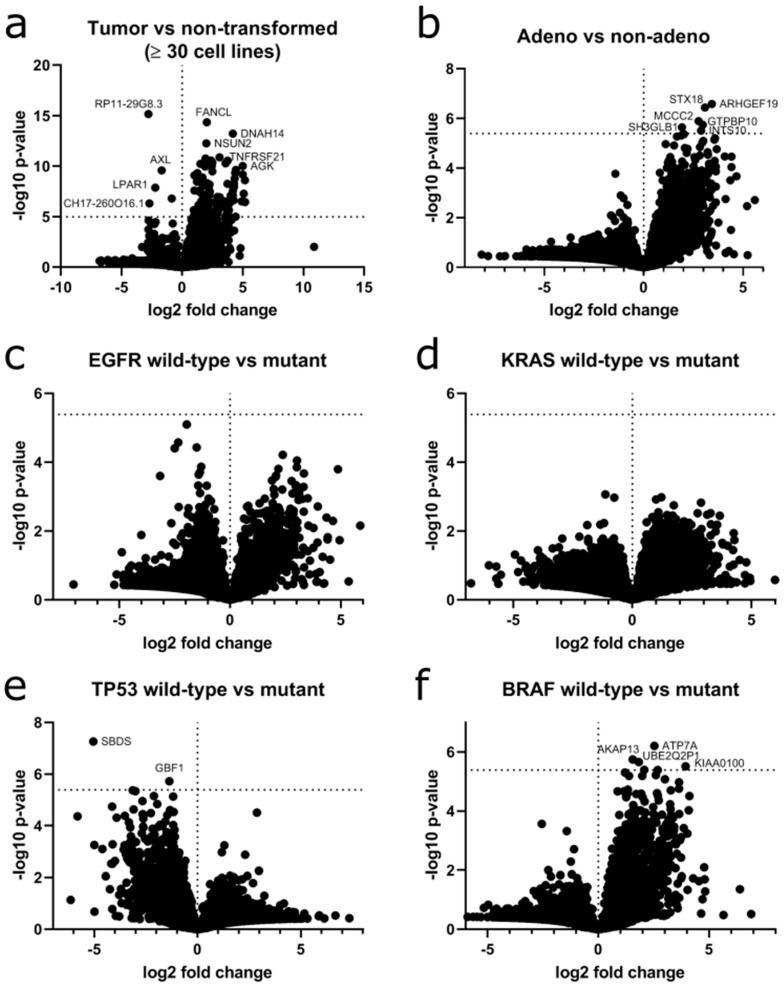
Volcano plots of circRNAs at gene level according to transformation status, histological subtype or genotype depicting fold change and statistical significance. (**a**) Differential circRNA expression in tumor-derived cell lines versus non-transformed cell lines. (**b**) Differential circRNA expression in adenocarcinoma cell lines versus non-adenocarcinoma NSCLC cell lines. (**c**) Differential circRNA expression in EGFR wild-type cell lines versus EGFR mutant cell lines. (**d**) Differential circRNA expression in TP53 wild-type cell lines versus TP53 oncogenic mutant cell lines. (**e**) Differential circRNA expression in KRAS wild-type cell lines versus KRAS mutant cell lines. (**f**) Differential circRNA expression in BRAF wild-type cell lines versus BRAF mutant cell lines. Vertical dotted line depicts a fold change of zero. Horizontal dotted line depicts *p*-value threshold for significance corrected for multiple testing. Gene names of genes with a significant fold change in circRNA expression are depicted where possible.

Next, we calculated the difference in circRNA expression according to transformation state, histological subtype and genetic background. When grouping cell lines according to their transformation status (NSCLC vs. non-transformed), the difference in circRNA expression of 128 genes reached statistical significance after correction for multiple testing, e.g., *circTNFRSF21* (Figure 9a).

Next, we grouped the tumor cell lines according to their histology (adenocarcinoma vs. non-adencarcinoma NSCLC, Figure 9b). A generally higher circRNA expression was found in adenocarcinoma. For 22 genes including *TALDO1*, *TMC5*, *MVP* and *FNDC3B,* this change in circRNA expression was significant. Lastly, we grouped the tumor cell lines according to their genotype as determined in the COSMIC cell line project [42]. We focused on prevalent mutations in lung cancer and compared mutant to wildtype cell lines. Cell lines harboring mutations in *EGFR* (Figure 9c) and *KRAS* (Figure 9e) did not show any significant changes. *TP53* mutant cells showed a higher expression of circRNAs with significance for *circSBDS*, *circGBF1*, *circATP6V1A* and *circVANGL1* (Figure 9d). Cell lines harboring *BRAF* mutations showed an overall lower circRNA expression with 10 genes reaching significance (Figure 9f). All differentially expressed circRNAs are listed in Supplementary Table S4. Overall, selected circRNAs correlate with the transformation status, the histology or the genotype of the lung cells in our study making them candidates for future evaluation of biomarkers due to their high stability.

### 2.9. circRNAs Were Efficiently Overexpressed in NSCLC Cell Lines with Laccase2 Introns

**Figure 10:**
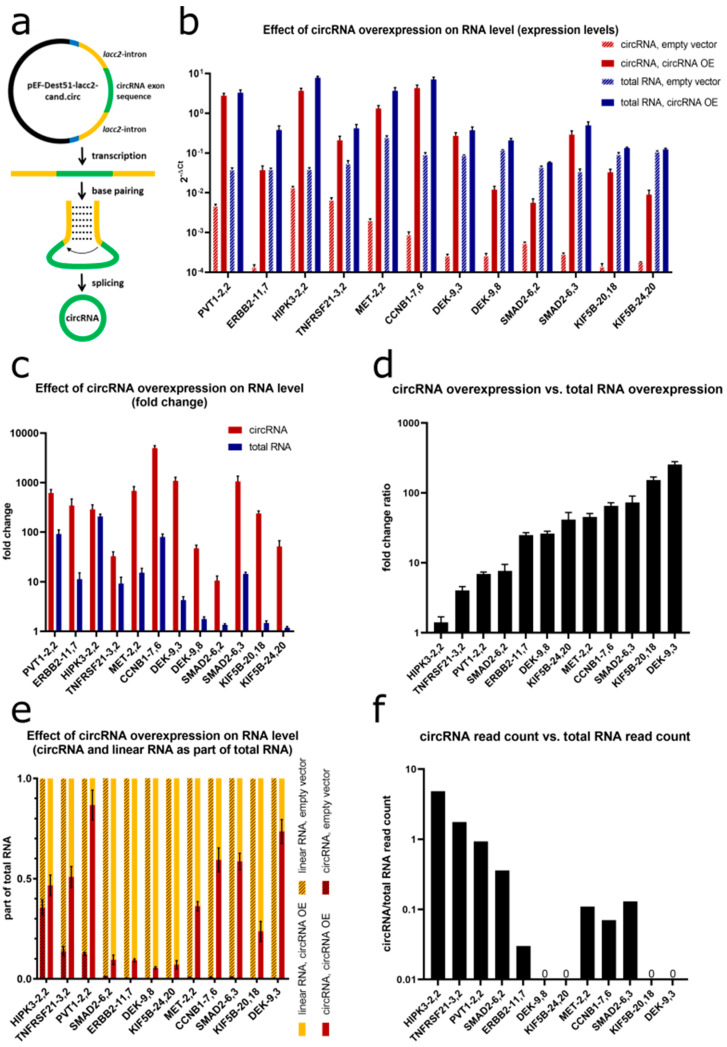
CircRNA overexpression (**a**) vector: pEF-Dest51-lacc2-cand.circ, the candidate circRNA exonic sequence (cand.circ) is flanked by laccase 2 introns (lacc2). Base-pairing between laccase2 introns enhanced backsplicing of the circRNA exonic sequence. (**b**–**e**) Effect of circRNA overexpression on circRNA and total RNA levels measured by RT-qPCR normalized to Cyclophilin A. Cell lines were transfected with the circRNA overexpression vector or empty vector as control. *n* ≥ 3. (**b**) Expression levels of circRNA and total RNA in controls and after overexpression depicted as 2^−ΔCt^. (**c**) Fold changes of circRNA and total RNA after overexpression compared to control. (**d**) Ratio between fold change of circRNA and fold change of total RNA after overexpression compared to control. (**e**) CircRNA and linear RNA as part of total RNA in controls and after overexpression. (**f**) Ratio between average circRNA and average total RNA read counts in RNA-Seq in the respective cell line used for overexpression. circRNA read count of zero in this cell line is depicted as 0.

To further investigate the phenotype of circRNAs in NSCLC cell lines, overexpression vectors for 12 candidate circRNAs were designed. We used the pcDNA3.1-Laccase 2 designed by Kramer et al. [43], which contained a multiple cloning site flanked by the minimal circularization inducing sequences of the laccase2 introns [43]. Due to base-pairing between reverse complementary sequences within the introns, backsplicing was efficiently facilitated (Figure 10a). By combining gateway and restriction site cloning, we constructed the circRNA expression vector pEF-Dest51-lacc2-cand.circ with cand.circ representing the exonic sequence of the respective candidate circRNA. We transfected cell lines with low endogenous circRNA expression with circRNA overexpression vector or empty vector control. RT-qPCR confirmed the successful overexpression of all 12 candidate circRNAs. Since total RNA comprised not only linear RNA but also circRNA, an increase in total RNA after circRNA overexpression was accordingly observed (Figure 10b). Overexpression levels for circRNAs ranged between 11-fold for *circSMAD2* (backsplice site connecting exons 6-2) and 4996-fold for *circCCNB1* (backsplice site 7-6) (Figure 10c). For all candidates, the fold changes for circRNA were higher than for total RNA derived from the gene. Hence, the fraction of circRNA in total RNA increased after overexpression (Figure 10d). The ratio between circRNA overexpression and total RNA overexpression was quite consistent for each circRNA between the replicates, but heterogeneous between the individual circRNAs. The highest ratio was found for *circDEK* (backsplice 9–3) increasing 1083-fold whereas total *DEK-9,3* only increased 4-fold leading to a circRNA/total RNA fold change ratio of 255. At the other end, *HIPK3*-2,2 showed the lowest ratio with 290-fold change for the circRNA vs. 208-fold change for the total RNA, resulting in an overall increase in the circular to total ratio of only 1.4. Due to the high variability in initial circRNA levels between the respective candidates and the different overexpression properties, the proportions of circRNA relative to total RNA after overexpression differed substantially among the circRNAs ranging between 6% for *circDEK* (backsplice site 9–8) and 86% for *circPVT1* (backsplice site 2–2) (Figure 10e).

Next, we analyzed whether the extent of the circRNA overexpression ratio (Figure 10d) correlated with the initial ratio of circRNA relative to total RNA (Figure 10f). Comparing the RNA-Seq read counts for the respective circRNAs and total RNAs in the FL3C dataset, we indeed found a negative correlation (*R* = −0,38) between the endogenous circRNA/total RNA read count ratio and the circRNA/total RNA ratio change upon overexpression. This indicated that for circRNAs which were present in a high fraction of the total RNA already endogenously, also the overexpression of circRNA and total RNA remained rather parallel (e.g., *circHIPK3-2,2*). For circRNAs which made up only a very small fraction of the endogenous total RNA, the overexpression largely increased the fraction of circRNA relative to total RNA (e.g., *circDEK-9,3*).

Overall, we successfully constructed vectors that efficiently and specifically overexpressed circRNAs at base-pair resolution.

### 2.10. circTNFRSF21 is Translated in Two Different Reading Frames Crossing the Backsplice Site Twice

**Figure 11:**
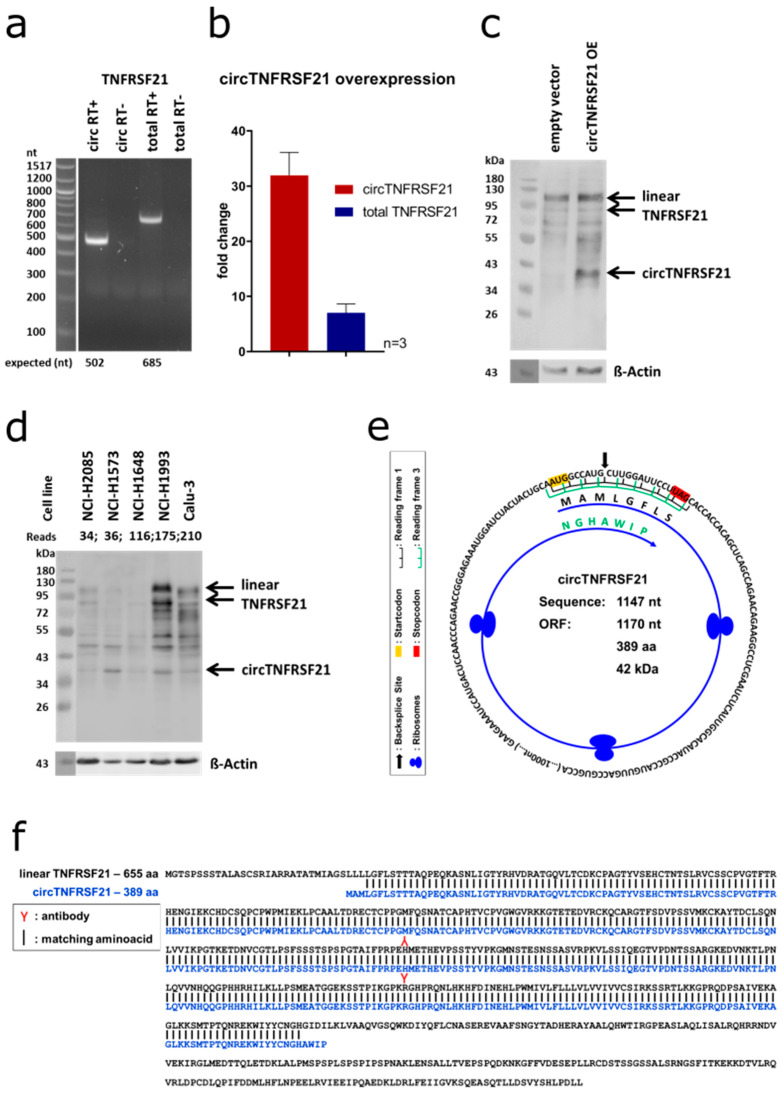
Translation of *circTNFRSF21*. (**a**) Validation of *circTNFRSF21* expression (circ) and total *TNFRSF21* expression (total). Test samples: “RT+”, negative controls: “RT-” lacking reverse transcriptase. Numbers represent expected fragment sizes in number of nucleotides. (**b**) Signal strength of bands after gel electrophoresis quantification (*n* = 3). (**c**) Effect of *circTNFRSF21* overexpression on protein synthesis. Representative western blot image after transfection. ß-Actin was used as loading control, 30 µg of total protein was loaded. (**d**) Endogenous protein expression of TNFRSF21. Representative western blot image. ß-Actin was used as loading control, 30 µg of total protein was loaded. (**e**) *circTNFRSF21* nucleotide sequence of 1147 nt and the predicted open reading frame (ORF) with 1170 nt. Start codon is marked in yellow, stop codon is marked in red. The frame crossing the backsplice site first is marked in black (reading frame +1), the frame after the second crossing of the backsplice site is marked in green (reading frame +3). (**f**) Amino acid sequence of TNFRSF21 derived from the linear mRNA (655 aa, black) and derived from the *circTNFRSF21* (389 aa, blue). The antibody used for western blot was raised against a region around Histidin-235 (red Y) which is included in the sequence of both proteins.

One circRNA with high abundance and high differential expression in tumor cell lines versus non-transformed cell lines was *circTNFRSF21*. We validated the expression of *circTNFRSF21* backsplice site linking exon 2 to exon 3 (2–3) by PCR (Figure 11a,b).

This circRNA showed no significant fold change after RNase R treatment, in contrast to a significant decrease in the total *TNFRSF21* RNA (linear and circular) and *Cyclophilin A* RNA expression levels (Supplementary Figure S1). Next, we overexpressed *circTNFRSF21* (Figure 11b) and performed western blotting for TNFRSF21. The full-length TNFRSF21 protein gave rise to two bands of respectively 80 and 120 kDa as expected and detected in both overexpressed and control samples in comparable amounts indicating unaltered endogenous TNFRSF21 protein expression.

Upon *circTNFRSF21* overexpression, we noticed a prominent additional band at approx. 42 kDa (Figure 11c). This band was also present endogenously in several cell lines which showed endogenous *circTNFRSF21* expression at the RNA level (Figure 11d). When assessing the coding potential of *circTNFRSF21* with the NCBI ORFfinder, we identified an open reading frame in *circTNFRSF21*, that crosses the backsplice site twice in two different reading frames (a schematic representation can be found in Figure 11e) and results in a protein of an estimated molecular weight of 42 kDa (Figure 11f). In conclusion, circTNFRSF21 could be translated into a 42 kDa protein.

### 2.11. circRNA Overexpression influences Colony Formation in NSCLC Cell Lines

To determine the influence of circRNAs on the colony forming capacity of lung cancer cells, we overexpressed several circRNAs in cell lines with low endogenous expression of the respective circRNA and compared their impact on colony formation to empty vector controls.

**Figure 12:**
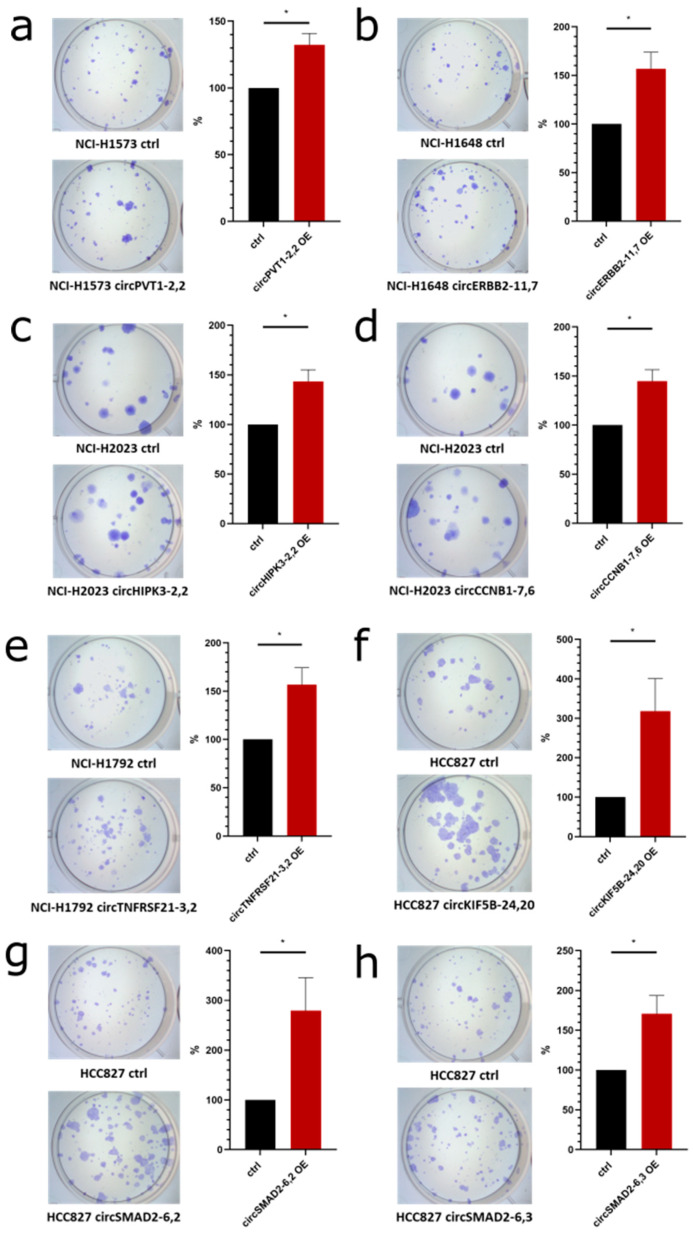
CircRNA overexpression impact on colony formation in lung cancer cells. Left: representative images of colonies after staining with crystal violet. Right: area covered by colonies after overexpression depicted in % of control, n ≥ 3. Cells were transfected with overexpression (OE) vector or empty vector as control (ctrl). (**a**) NCI-H1573 cells transfected with *circPVT1*-2,2 OE vector. (**b**) NCI-H1648 cells transfected with *circERBB2*-11,7 OE vector. (**c**) NCI-H2023 cells transfected with *circHIPK3*-2,2 OE vector. (**d**) NCI-H2023 cells transfected with *circCCNB1*-7,6 OE vector. (**e**) NCI-H1792 cells transfected with *circTNFRSF21*-3,2 OE vector. (**f**) HCC827 cells transfected with *circKIF5B*-24,20 OE vector. (**g**) HCC827 cells transfected with *circSMAD2*-6,2 OE vector. (**h**) HCC827 cells transfected with *circSMAD2*-6,3 OE vector. *T*-test: not significant (n.s.), *p*-value < 0.05 (*).

We started with the overexpression of *circPVT1* (backsplice site 2–2) (Figure 12a), which is a known oncogenic circRNA [44,45,46], which resulted in a significant increase in colony formation as expected (Figure 12a). Furthermore, overexpression of *circERBB2* (backsplice site 11–7) which emanated from a known oncogenic driver gene in lung cancer [47] led to a significant increase in colony formation (Figure 12b).

As an example of a gene that had not been connected to cancer before (non-CGC gene), we selected *circHIPK3* which was highly expressed and showed a very high ratio of circular/total RNA expression. Overexpression of *circHIPK3* (backsplice site 2–2) also significantly increased colony formation (Figure 12c). Since *circCCNB1* (backsplice site 7–6) was derived from a gene involved in cell cycle control [48], we selected it and found a significant increase in colony formation upon overexpression (Figure 12d). Also for the overexpression of *circTNFRSF21* (backsplice site 3–2), which was differentially expressed in tumor vs. non-transformed cell lines (Figure 9a), we observed a significant increase in colony formation (Figure 12e).

Also, we analyzed the correlation of circRNA expression with the proliferation capacity which we had determined for all lung cell lines in our FL3C panel (Figure 8). *CircKIF5B* (backsplice site 24–20) showed a positive correlation of 0.29 with proliferation. In the colony formation assay, the overexpression of *circKIF5B* (backsplice site 24–20) caused a significant increase (Figure 12f). Moreover, we identified a highly positive correlation of two circRNAs generated from the *SMAD2* gene, a known cancer gene listed in CGC. Overexpression of *circSMAD2* (backsplice site 6–2, *R* = 0.37, Figure 12g) and *circSMAD2* (backsplice site 6–3, *R* = 0.35, Figure 12h) also showed a significantly increased colony formation.

In summary, these results demonstrate the functional impact of selected circRNAs identified in our resource validating its usefulness to find circRNAs for future functional studies to unravel their role in lung cancer.

## 3. Discussion

In our study, we created a comprehensive resource of the circRNA landscape in lung cells from 175 transcriptomes of rRNA-depleted RNA with 2.8 million backsplicing reads identifying and quantifying circRNAs. The depletion of rRNA allowed us to sequence simultaneously both the circRNA and the linear RNA transcriptome across the cell lines. In comparison, we found significantly and strongly decreased circRNA read counts in public polyA-enriched RNA-Seq datasets, which was not surprising given that circRNAs do not contain a polyA tail at their 3′-end. The frequently used TCGA and CCLE databases are both prepared using polyA selection [30,40]. Hence, while these are highly valuable tools for the analysis of polyadenylated RNAs (e.g., mRNAs), these should be used with caution for circRNAs. Another approach is the use of the exonuclease RNase R to digest most of the linear RNA by exonucleolytic degradation. This method will specifically enrich circRNA reads, but prevents a comparison between circRNA and total (linear) RNA since the linear RNA is depleted. Finally, exome capture was designed to detect both circRNA and linear RNA in samples with poor quality RNA [49]. Although this method resulted in a high amount of reads for both circRNA and linear RNA [16], the usage of probes might create a sequence bias and novel transcripts lacking complementarity to the probes cannot be detected.

We analyzed the data both at the gene level (grouping all the circRNAs arising from the same gene) and at the backsplice level (for every unique circRNA, i.e., every backsplice site defined by two linked exons). Strikingly, 14.1% of the genes formed circRNAs in ≥90% of the 60 cell lines, whereas only 0.7% of individual circRNAs were found in ≥90% of the cells. A similar pattern emerges from the analysis of the correlation between the circRNA relative to the total (linear) reads from the same locus: the correlation was much higher at the gene level (median 0.27) than at the backsplice level (median 0.06). This observation could point to a hypothesis that in general, the formation of circRNAs from a gene is more “important” and hence more homogeneous and more frequently correlated than forming a specific circRNA from the respective gene―which may be subject to future studies to unravel the functional importance of different circRNAs produced from the same locus.

Our data also illustrates a high cell line specificity for most circRNAs. This is corroborated by a comparison with MiOncoCirc [16], which reported a subset of 589 out of 12397 genes (4.8%) giving rise to circRNAs in >90% of the cell lines tested. The higher fraction (14.1%) in our FL3C dataset is likely explained by the inclusion of different cancer entities in MiOncoCirc, while our dataset contains only lung cells and is hence derived from a more homogeneous tissue background.

When looking at the acceptor and donor exons, we noticed that the first and last exons were almost excluded from circRNA formation in comparison to the second and second-to-last exons, respectively. The predominance of the second exon as an acceptor exon is in accordance with previous data by Ragan et al. [50]. Mechanistically, the lack of acceptor exons in first exons may point towards the post-transcriptional nature of backsplicing [10], which is likely blocked by co-transcriptional 5′-capping. The last exon cannot act as donor exon since its 3′-end is cut and polyadenylated rather than spliced.

Focusing on the number of circRNAs originating from a single gene, *BIRC6* showed the highest number of circRNA isoforms in the FL3C dataset (418), which was in agreement with MiOncoCirc reporting more than 500 isoforms of *circBIRC6* [16]. The gene with the highest circRNA read counts in our data was *CYP24A1*, which has also previously been identified as the most highly expressed circRNA in the single A549 cell line [51].

Unsupervised hierarchical clustering of the top 100 most highly expressed circRNAs both at the gene level and at the backsplice level resulted in a separate cluster for the non-transformed cell lines, whereas this separation was less prominent between adenocarcinoma and non-adenocarcinoma NSCLC cell lines. Hence, circRNA expression levels could distinguish different cell types, which is in agreement with a previous study in hematopoietic cells [52].

The overall correlation between circRNA expression and total (linear) RNA expression was neutral or positive, with a stronger positive correlation at the gene level than at the backsplice level, while negative correlations were very rare and only weak. Given that the linear primary RNA transcript is the precursor of both the mature mRNA and the circRNA, this trend to a positive correlation may not be unexpected. Several previous studies reported a lack of a significant correlation between circular and linear RNA. This difference may be explained largely by multiple reasons: first, several of these studies compared circular and linear RNA ratios in different heterogeneous tissues or different developmental stages [51,53], while our study analyzed the correlation of circular and linear RNA in a more homogeneous of cell lines derived from the same organ and most of them from the same tumor subtype―lung adenocarcinoma. Hence, there may be differential efficiency of RNA circularization between different tissues, but this may be less pronounced in the same tissue. Second, another study based the correlation analysis of circular and linear RNAs on only highly abundant circRNAs (279 out of 15,223) [54], while our study included 148,811 circRNAs (all) or 5232 circRNAs with expression in more than half of the cell lines. Lastly, multiple previous studies were based on RNA-seq of polyA-enriched RNA, while we used rRNA-depleted RNA which may also cause a different representation of the circRNAs. Nonetheless, also our data states that the correlation between circular and total (linear) RNA is weak in most cases.

The differences in correlation for the different circRNAs from the same parental mRNA might be explained by the different elongation rates of RNA polymerase II (reviewed in [55]). The production rate of circRNAs is positively correlated with the transcription elongation rate [10]. A second factor influencing the circRNA biogenesis is the availability of the splicing machinery [56] and the efficiency of canonical splicing [8]. When splicing is less efficient, the RNA has more opportunities to circularize upon itself which eventually leads to backsplicing. This circularization might be influenced by flanking introns containing complementary sequences [11], RNA binding proteins [13,14] or RNA editing [11]. Taken together, both the transcription elongation rate and splicing contribute to the formation of circRNA, and might explain the differences in circRNA expression levels between circRNAs originating from the same mRNA.

The known cancer genes (CGC) [42] produced on average more circRNAs in comparison to non-CGC genes. There are two possible explanations: either these CGC genes are more abundantly transcribed in comparison to non-CGC genes and thus there are more opportunities for circRNAs to be formed as a by-product from canonical splicing. Or these circRNAs are produced to fulfill an oncogenic function in the cell―which will be a relevant topic for elucidation in the future.

When correlating circRNA expression to the proliferation rate of the cell lines, there was a larger subset of circRNAs that were only expressed in the slow and a smaller subset of circRNAs that were only expressed in fast proliferating cells. This result was in accordance with previous reports [15,16] and warrants future research into a functional role for the differentially regulated circRNAs. So far, there is no verified explanation for the general decrease in circRNA expression in fast proliferating cells, but possibly the stable circRNAs could be diluted by cell division [15]. Another possibility is a deregulation of the splicing machinery thus influencing the production of circRNAs. However, more research is needed to elucidate the exact regulatory mechanism in highly proliferating cells.

Adenocarcinoma cell lines showed a higher expression of circRNAs in comparison with non-adenocarcinoma NSCLC cell lines. This may in part be due to a difference in the average proliferation rate of the cells [15]. The seven non-LUAD cell lines have an average proliferation rate of a 6.0-fold increase in cell count after 72 h, in comparison to a 3.3-fold increase for the 50 LUAD cell lines (significant: *T*-test *p* = 0.0014). Among the genes with a significant change in circRNA expression, some had been previously identified as markers for LUAD: *TALDO1* [57], *TMC5* [58], *MVP* [59] and *FNDC3B* [60]. This confirms that our cell line panel is able to recapitulate biomarker patterns at the circRNA level which were previously established at the linear mRNA level for different NSCLC histological subtypes. When comparing *TP53* wild-type versus *TP53* mutant cell lines, there were two circRNA-producing genes that showed a significant change: *SBDS* and *GBF1*, with *SBDS* having previously been linked to *TP53* [61].

Comparing tumor cells to non-transformed cell lines identified a number of significantly differentially expressed circRNAs―including *circTNFRSF21*. Overexpression of this circRNA gives rise to an additional band matching in size to an open reading frame (ORF) in *circTNFRSF21* which crosses the backsplice site twice in two different reading frames. The translation of circRNAs has been described previously [62]. Given the lack of a 5′-cap structure, mechanisms like an IRES sequence [63] or m6A methylation could initiate translation [64]. Eukaryotic cells are capable of translating circRNAs according to a rolling circle mechanism [65], which is fitting with the ORF in *circTNFSF21* crossing the backsplice site twice. Further research is warranted to find out the translation initiation mechanism of *circTNFRSF21* and its functional role in lung cancer.

We showed that overexpression of circRNAs is possible, using an adapted method that was originally described by Kramer et al. [43], using the highly complementary sequence of the *Laccase2* introns form *Drosophila melanogaster*. The base-pairing between the intronic sequences brings the donor and acceptor exon into close proximity to each other, thus creating favorable conditions for circularization [43,66]. However, the efficiency in overexpressing circRNAs was heterogeneous. This reflects in part the endogenous ratio of circular to total RNA, but might also be explained by the role of other factors like the different transcription efficiency [10].

Overexpression of circRNAs allowed us to study their phenotype and influence on colony formation. First, circRNAs originating from known cancer genes (CGC-listed genes) also showed an oncogenic effect and their overexpression in low endogenously expressing cell lines led to increased colony formation, e.g., for *circPVT1*, which corroborates previous studies [45,46]. Second, we identified circRNAs from known cancer genes and from non-cancer genes to influence lung cancer cell colony formation. Altogether, these data indicate that further functional studies are warranted to unravel the oncogenic potential of these circRNAs.

In summary, our study provides a deep insight into the expression of circRNAs in lung cell lines. Next to our findings on global circRNA properties like correlation with linear RNA, exon distribution, distinction between non-transformed and tumor cell lines by PCA, enrichment in cancer genes and association with cell proliferation and genotypes, we provide individual examples of circRNAs governing the colony formation potential of lung cancer cells as well as *circTNFRSF21* being translated with two reading frames across the backsplice site. For the future, our resource can be widely used to identify new circRNAs in lung cancer, to find cell lines with endogenous expression of specific circRNAs for further experiments, to search for genes with a particularly high or low circularization rate, to select circRNAs correlated with cell proliferation for mechanistic studies or to identify circRNAs associated with certain genotypes in lung adenocarcinoma.

## 4. Materials and Methods

### 4.1. Cell Culture

All cell lines were purchased from American Type Culture Collection (ATCC, Manassas, VA, USA) and were maintained in ATCC-modified RPMI-1640 medium (cat#A1049101, Life Technologies, Carlsbad, CA, USA) at 37 °C and 5% CO_2_ in cell culture dishes (TPP, Trasadingen, Switzerland) [67]. At maximum 80% confluence, cells were detached using 0.25% trypsin (Life Technologies, Carlsbad, CA, USA) and splitted 1:3–1:5 dependent on the cell line. Cells were regularly tested for mycoplasma contamination. The total FL3C database contains 50 lung adenocarcinoma cell lines (LUAD), seven non-LUAD NSCLC cell lines and three non-transformed cell lines (Supplementary Table S1). Genotype data was downloaded from the COSMIC Cell line project, v90 [42].

The proliferative capacity of the cell lines was determined as follows: 150,000 cells per well were seeded into a 6-well plate and trypsinized after 24 h, 48 h and 72 h, respectively (2-wells per timepoint). At each timepoint, the cells were counted in duplicate. Ratios between the starting amount of cells (150,000/well) and cell count after 24, 48 and 72 h were determined. The increase in ratios represented the proliferative capacity of the cell line and is provided in Supplementary Table S1 (72 h).

### 4.2. RNA-Seq Library Construction and circRNA Analysis

RNA was isolated with the RNeasy mini kit (Qiagen, Hilden, Germany). An on-column DNase I digest was performed with the DNase I RNase-free DNase set (Qiagen). From 5 µg total RNA, ribosomal RNA (rRNA) was depleted with the RiboZero Gold Kit H/R/M (Illumina, San Diego, CA, USA). The RNA library was constructed with the SureSelect Strand-Specific RNA library prep for the Illumina Multiplexed Sequencing Protocol (Illumina) and sequenced with the HiSeq4000 paired end 100 bp kit (Illumina). All kits were used according to manufacturers’ instructions.

Raw reads were mapped using Tophat2 [68,69] with the following parameters: -a 6 -m 2 -g 1 -p 16. Reads that were not aligned with Tophat2 were filtered and aligned by Tophat2 fusion using the following parameters: - fusion search - p 15 - keep fasta order - bowtie1 - no coverage search. The circExplorer2 [70] pipeline was used to identify circRNAs using standard parameters. CircRNAs were length-normalized using the fragment-length as determined by DESeq [71]. All reads were mapped to the Ensembl GRCh38 gene set.

The number of reads mapping to the backsplice side were used to calculate the circRNA expression level, while the sum of the total linear reads mapping to the parental gene was used to measure the total (linear) RNA expression level. The circular/total ratio was calculated by dividing the normalized backsplice reads (normalized to library size) by the linear FPKM. All statistical analyses were performed in R and the R-packages described (v3.6.0) [72].

### 4.3. RNA Isolation for RT-(q)PCR

Cells were washed with 1x PBS (Sigma-Aldrich, St. Louis, MO, USA) and lysed in Tri-reagent (Sigma-Aldrich). RNA was isolated according to the manufacturer’s instructions. DNA was digested with DNase I (Roche, Mannheim, Germany) for 30 min at 37 °C. Reverse transcription was performed on 1 µg of RNA with RevertAid Reverse Transcriptase (ThermoFisher, Schwerte, Germany) and random hexamer primers (ThermoFisher).

### 4.4. PCR and Quantitative Real-Time PCR

cDNA was amplified using the Q5 high fidelity polymerase (New England Biolabs, Ipswich, MA, USA) following the manufacturer’s instructions according to the following program: an initial incubation of 30 s at 98 °C, followed by 30 cycles of 10 s 98 °C, 30 s 58 °C and 45 s 72 °C, followed by a final incubation of 5 min at 72 °C in a Bio-Rad T100 Thermal Cycler. Next, the PCR-reaction was mixed with DNA loading dye (New England Biolabs) and separated on a 1% or 2% agarose gel containing SybrSafe (Thermo Fisher) and run in 1× TAE (40 mM Tris Base, 20 mM acetic acid, 1.27 mM EDTA, pH 8.3). The gel was imaged with the Gel iX20 imager (Intas, Göttingen, Germany). Band quantification was performed with Image Studio Lite software (Licor, Lincoln, NE, USA).

The expression of the circRNA and its corresponding total RNA were determined via RT-qPCR using 2× Power SybrGreen Master Mix (Applied Biosystems, Foster City, CA, USA). Reactions were run in triplicate on the Applied Biosystems StepOnePlus cycler using the following program: 10 min incubation at 95 °C prior to 40 cycles of 15 s at 95 °C and 30 s at 60 °C. *Cyclophilin A* was used as housekeeper gene for normalization. The relative expression was determined by the 2^−ΔΔCt^ method using average Ct values [73]. All primer sequences for PCR, RT-qPCR and cloning are listed in Supplementary Table S5.

### 4.5. RNase R Treatment

One µg of total RNA was treated with 10 U of RNase R (Lucigen, Middleton, WI, USA) in 10 µL of total volume with 0.2 µL 50 mM MgCl_2_ in PCR Single Cap 8er-SoftStrips reaction tubes for 60 min at 37 °C in a T100 Thermal cycler (Bio-Rad, Feldkirchen, Germany) according to manufacturer’s instructions. The reaction was prepared in parallel in duplicates. After incubation, one reaction mix was used for the RT-PCR and the second reaction served as non-enzyme control (no reverse transcriptase was added to this reaction). Protocols were further executed as described in Section 4.3 and Section 4.4.

### 4.6. Cloning

First, the minimal Laccase2 intronic sequence, including a multiple cloning site (MCS), from the pcDNA3.1(+) Laccase2 MCS Exon Vector (Addgene, Cambridge, MA, USA) [43] was amplified and inserted into the pDONR221 vector by BP Gateway cloning (ThermoFisher).

Second, the complete sequence of the circRNA was amplified by PCR (see above) followed by sequencing to assess which exons were included. The amplicons were separated on an agarose gel, the target band was purified using the GeneJET Gel extraction kit (ThermoFisher) and the bands were sequenced with SupremeRun Tube Sanger Sequencing (GATC Eurofins, Ebersberg, Germany).

Third, the target sequence was amplified by Q5-PCR (see above), separated on an agarose gel and purified. The circRNA sequence was inserted into the MCS using restriction site cloning with either PacI and AscI or PstI-HF and AscI (all from New England Biolabs). Next, the entry clones were recombined into the pEF-DEST51 Gateway expression vector (ThermoFisher) using the LR Clonase II enzyme mix (ThermoFisher) according to manufacturer’s instructions.

Lastly and importantly, to delete the remaining sequences of the MCS at both ends of the circRNA target sequences, a two-step PCR protocol was used. With a first PCR, the circRNA target sequence was amplified in two parts using overlapping primer pairs lacking the MCS sequence. In a second step, the outer primers were used to create the full target sequence. This was inserted into the expression vector by restriction enzyme cloning.

### 4.7. Colony Formation Assay

Cells were seeded in a 6 cm cell culture dish (TPP) and a 12-well plate (Greiner Bio-One, Kremsmünster, Austria) and allowed to attach overnight. A 1000–2000/well cells were seeded in the 12-well plate for colony formation assay, 250,000 cells were seeded in the 6 cm dish for RNA isolation. Next day, the cells were transfected with 1 µg (12-well) or 5 µg (6 cm culture dish) of plasmid using 1 mg/mL polyethylenimine (PEI) (Polysciences Inc., Warrington, PA USA) in a 1:3 ratio in OPTI-MEM (Gibco, ThermoFisher, Carlsbad, CA, USA) according to the manufacturer’s instructions. The empty vector was used as negative control.

After 48 h, cells from the 6 cm culture dish were lysed and RNA was isolated as previously described. Reverse transcription and quantitative real-time PCR was used to determine the level of overexpression of both the circRNA and the corresponding total (linear) RNA.

For the colony formation assay, cells in the 12-well plate were allowed to grow during 8–17 days, depending on the proliferation rate of the cell line (NCI-H1573: 10 days, NCI-H2023: 15–17 days, HCC827 and NCI-H460: 8–10 days). The medium was changed every 4 days during this period. Afterwards, cells were washed with 1× ice-cold PBS (Sigma) and fixed with 100% methanol (Carl Roth, Karlsruhe, Germany) during 10 min at −20 °C. Cells were stained with 0.5% crystal violet (Carl Roth) dissolved in 25% methanol in ddH_2_O during 10 min at room temperature. Remaining stain was removed by washing with ddH_2_O and the plate was air-dried overnight. The wells were imaged with a stereo Discovery V8 microscope (Carl Zeiss AG, Oberkochen, Germany) equipped with a 0.3× objective and 1.0× zoom and colony areas were determined using ImageJ [74].

### 4.8. Western Blotting

Cells were seeded in a 6 cm dish and transfected as described in 4.7. After 48 h, cells were lysed on ice in 1× RIPA buffer (150 mM NaCl, 1% Triton X-100, 0.5% Na-deoxycholate, 0.1% SDS, 50 mM Tris pH8, freshly supplemented with Complete protease inhibitor cocktail (cat#04693159001, Roche, Basel, Switzerland) and spun down for 15 min at 12,000× *g* at 4 °C. The lysate was transferred to a new tube. The protein concentration was measured with the BCA assay (Sigma, Darmstadt, Germany). Thirty µg of total protein was loaded on a 10% SDS-PAGE gel and run for 1 h at 120V. Next, proteins were semi-dry blotted onto a nitrocellulose membrane (1 h 20 min at 0.07 A). The membrane was blocked in 5% skimmed milk during 1 h at room temperature. The membrane was incubated overnight at 4 °C with the primary antibody for TNFRSF21 (DR6, clone E8D2I Rabbit mAb, #93026, Cell Signaling, Leiden, The Netherlands) in a 1:1000 dilution in 5% BSA in 1× TBS (24.7 mM Tris base, 137 mM NaCl, 2.68 mM KCl, pH 7.4) with 0.1% Tween-20. After washing with 1× TBS with 0.1% Tween-20, the membrane was incubated with the secondary antibody (Peroxidase-AffiniPure Goat Anti-Rabbit IgG, Dianova, Hamburg, Germany, 1:5000 dilution in 5% BSA in 1× TBS with 0.1% Tween-20) during 2 h at room temperature. After washing, bands were detected with SuperSignal West Pico Chemiluminescent Substrate (ThermoFisher) and visualized with the Intas ECL Chemocam Imager (Intas). As loading control, membranes were stained for β-Actin (clone AC74, β-actin mouse mAb, Sigma) in a 1:10000 dilution in 5% BSA in 1× TBS with 0.1% Tween-20, incubated for 2 h at room temperature. After washing, the membrane was incubated with the secondary antibody for 2 h at room temperature (Peroxidase-AffiniPure Goat Anti-Mouse IgG, Dianova, 1:10,000 dilution in 5% BSA in 1× TBS with 0.1% Tween-20).

### 4.9. Statistics

Statistics were performed in Excel (Microsoft, Redmond, WA, USA) and GraphPad Prism (version 8, GraphPad Software, San Diego, CA, USA). Statistical significance was assessed with a two-sided *T*-test after performing an *F*-test to test scedasticity. Experiments were always performed at least in biological triplicates. The RT-qPCR was performed in three and colony assays were performed in three to six technical replicates per biological replicate. Colony assay replicates with a cell colony number of zero were excluded from further calculations. The correlations per histology or genotype were performed by multiple *T*-tests (*T*-test per row) with Bonferroni correction for multiple testing. Groups with a mean value of zero were excluded from statistical analysis.

## 5. Conclusions

In this study, we present a comprehensive map of the circRNA landscape in the FL3C panel of 60 lung cell lines based on 2.8 million backsplice reads derived from deep sequencing of rRNA-depleted RNA. Most circRNAs show high cell line specificity and generally a positive correlation between circRNA and total (linear) RNA expression levels. Known cancer genes tend to produce more circRNAs. CircRNA patterns can be used for classification and clustering of cell lines or reflect tumor histology, genotype and proliferation rate. Correlation of cell proliferation with circRNA expression can identify circRNAs whose overexpression has a significant impact on colony formation of lung cancer cells. Moreover, *CircTNFRSF21* can be translated into a 42 kDa protein crossing the backsplice site in two different reading frames. In conclusion, this lung cell line dataset advances our understanding of circRNA expression and will enable future research on the function and relevance of circRNAs in lung cancer.

## Supplementary Materials

The following are available online at https://www.mdpi.com/2072-6694/12/5/1091/s1, Supplementary Table S1: Overview of the used cell lines; Supplementary Table S2: Full circRNA dataset at gene level; Supplementary Table S3: Full circRNA dataset at backsplice level; Supplementary Table S4: Differentially expressed circRNAs; Supplementary Table S5: Primer sequences (subtables for primers used for PCR, RT-qPCR, cloning and mutagenesis, all sequences in 5′ to 3′ direction). Supplementary Figure S1: Resistance of candidate circRNAs to exonucleolytic degradation by RNase R; Supplementary Figure S2: Validation of proliferation-correlated circSMAD2-6,2 in fast vs. slowly proliferating cell lines with RT-qPCR. The first two cell lines (NCI-H1944, NCI-H1993) were fast proliferating (≥5-fold increase in cell count in 72 h), the last two cell lines (HCC827, NCI-H2347) were slowly proliferating (≤3-fold cell count increase in 72 h). Expression levels of circSMAD2-6,2 and total SMAD2-6,2 are depicted as % of maximum expression level (in NCI-H1993). *T*-test: not significant (n.s.), *p*-value < 0.05 (*), *p*-value < 0.01 (*), *p*-value < 0.001 (**). Below the graph, the fold change of average relative expression level between the groups is shown.
