# Supplementary material for: The Circular RNA Landscape of Non-Small Cell Lung Cancer Cells
Source: Cancers (Basel). 2020 Apr 28;12(5):1091. doi: 10.3390/cancers12051091 (PMC7281449; doi:10.3390/cancers12051091)
Supplement: Supplementary file 1 [file cancers-12-01091-s001.zip › Suppl Figure S1.pdf]

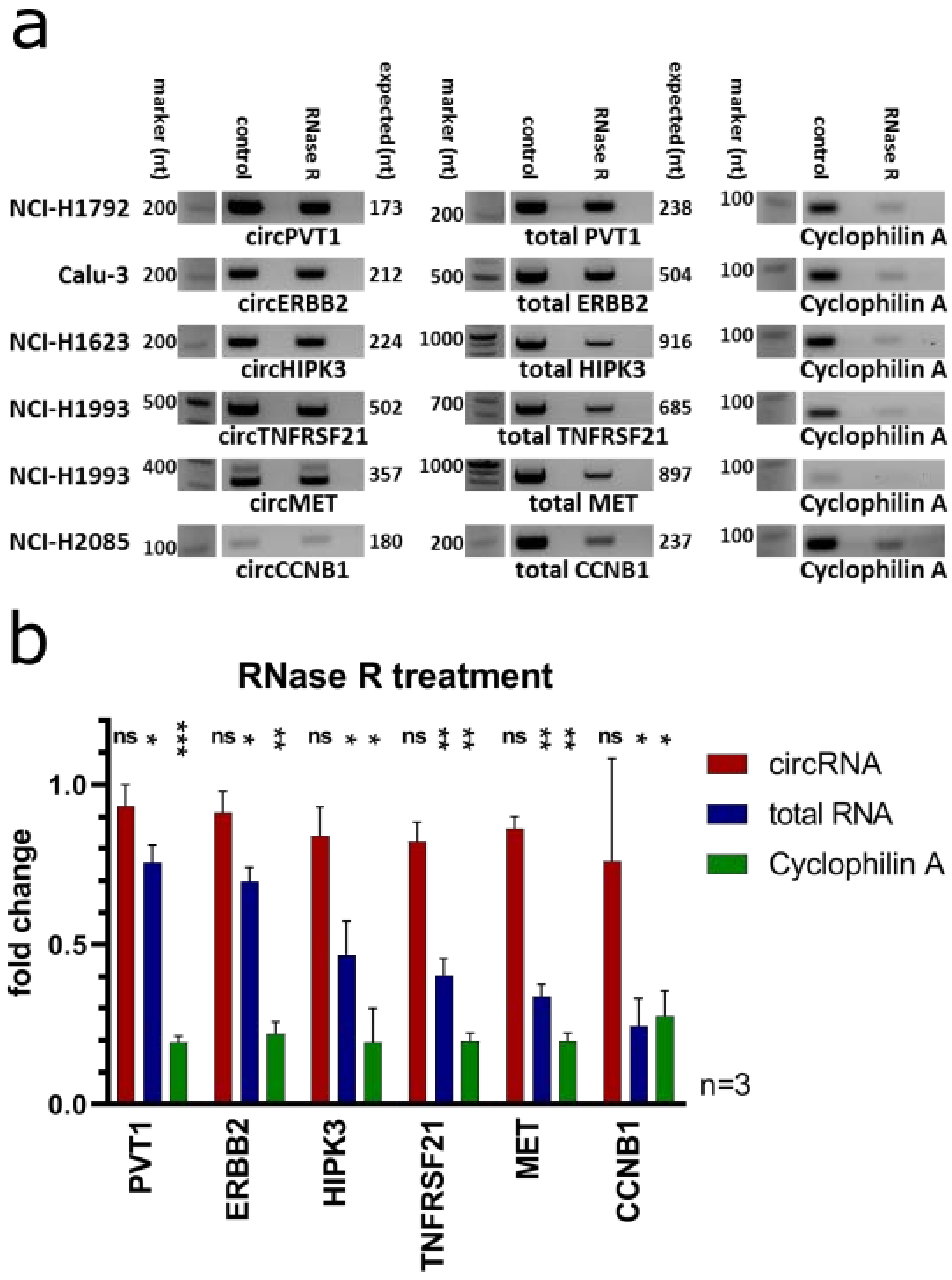

Supplementary Figure S1: Resistance of candidate circRNAs to exonucleolytic degradation (a) Representative images of bands after RNase R treatment vs. control for circular and total PVT1, ERBB2, HIPK3, TNFRSF21, MET and CCNB1 and total Cyclophilin A as a control after gel electrophoresis. RNase R reaction was performed on 2 µg of RNA, half of this reaction was used for RT-PCR, other half served as control reaction (no reverse transcriptase added to the reaction). Marker sizes are depicted on the left, expected band sizes on the right. (b) Signal strength of the bands after gel electrophoresis was quantified (n=3). Shown is the fold change in signal strength compared to control for circRNA, total RNA and Cyclophilin A respectively. T-test not significant (n.s.), p-value <0,05 (*), p-value <0,01 (**), p-value <0,001 (***).
