# Supplementary material for: The Circular RNA Landscape of Non-Small Cell Lung Cancer Cells
Source: Cancers (Basel). 2020 Apr 28;12(5):1091. doi: 10.3390/cancers12051091 (PMC7281449; doi:10.3390/cancers12051091)
Supplement: Supplementary file 1 [file cancers-12-01091-s001.zip › Suppl Figure S2.pdf]

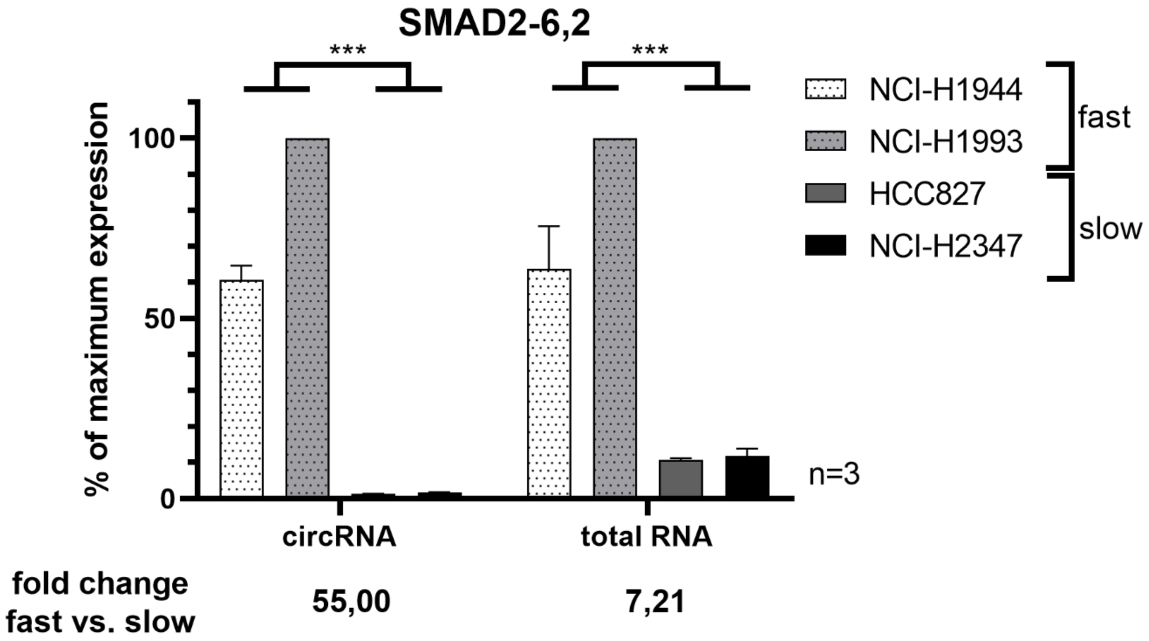

Supplementary Figure 2. Validation of proliferation-correlated circSMAD2-6,2 in fast vs. slowly proliferating cell lines with RT-qPCR. The first two cell lines (NCI-H1944, NCI-H1993) were fast proliferating (≥ 5-fold increase in cell count in 72h), the last two cell lines (HCC827, NCI-H2347) were slowly proliferating (≤ 3-fold cell count increase in 72h). Expression levels of circSMAD2-6,2 and total SMAD2-6,2 are depicted as % of maximum expression level (in NCI-H1993). T-test: not significant (n.s.), p-value<0,05 (*), p-value<0,01 (**), p-value<0,001 (***). Below the graph, the fold change of average relative expression level between the groups is shown.
