# Supplementary material for: The Circular RNA Landscape of Non-Small Cell Lung Cancer Cells
Source: Cancers (Basel). 2020 Apr 28;12(5):1091. doi: 10.3390/cancers12051091 (PMC7281449; doi:10.3390/cancers12051091)
Supplement: Supplementary file 1 [file cancers-12-01091-s001.zip › Suppl Western Blots.pdf]

Figure 11c: TNFRSF21 overexpression

Replicate 1: marker for TNFRSF21

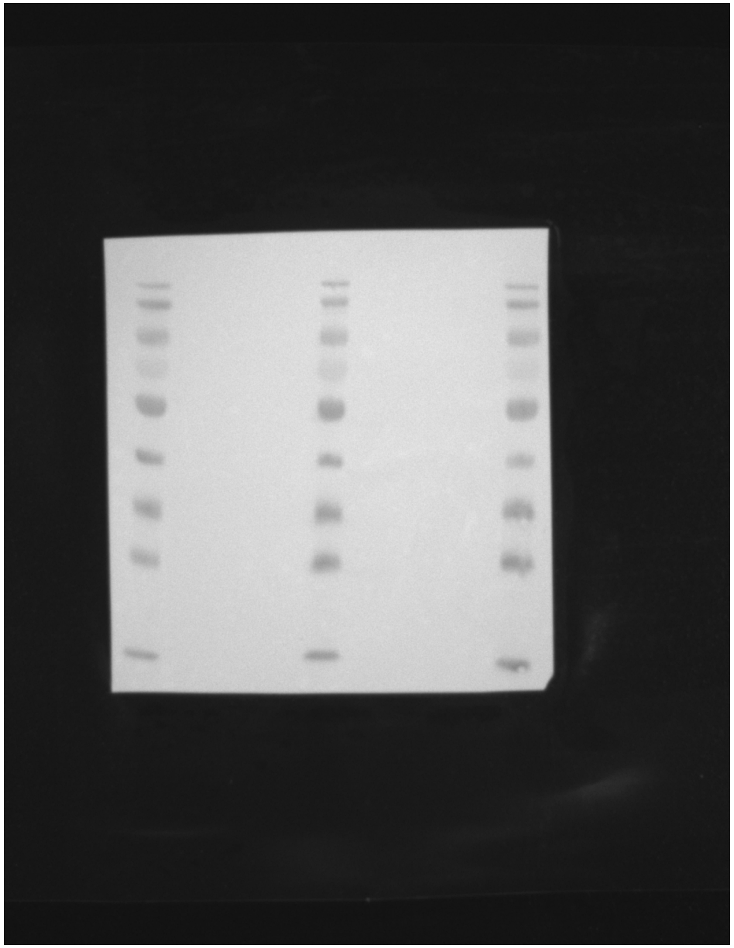

Figure 11c: TNFRSF21 overexpression

Replicate 1: TNFRSF21

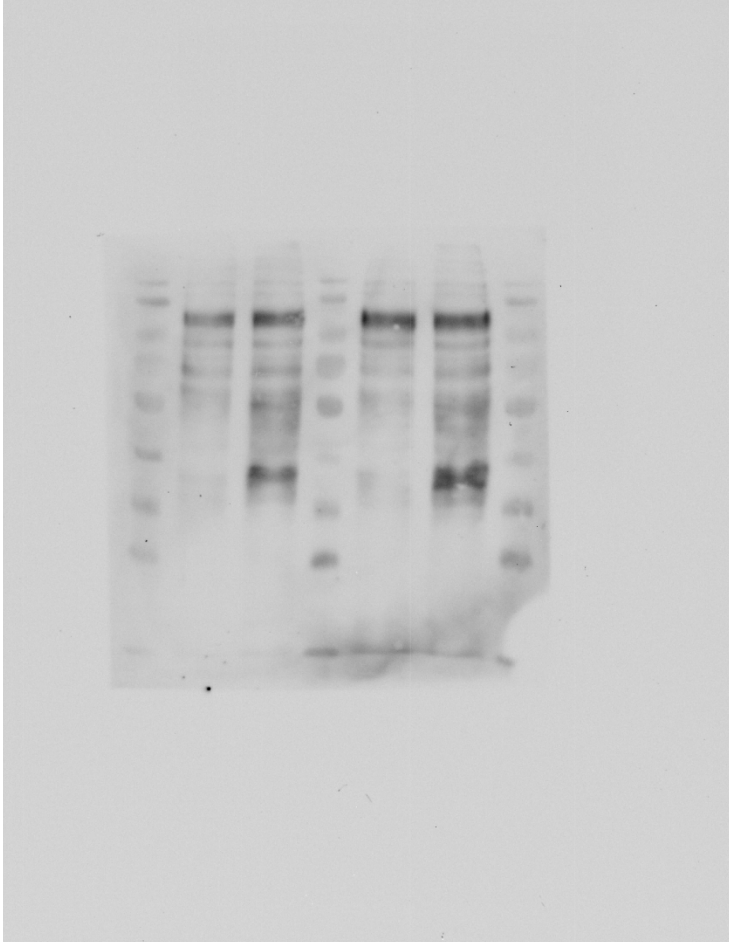

From left to right: empty vector control (30µg protein), TNFRSF21 overexpression (30µg protein), empty vector control (50µg protein), TNFRSF21 overexpression (50µg protein)

Figure 11c: TNFRSF21 overexpression

Replicate 1: marker for ß-Actin

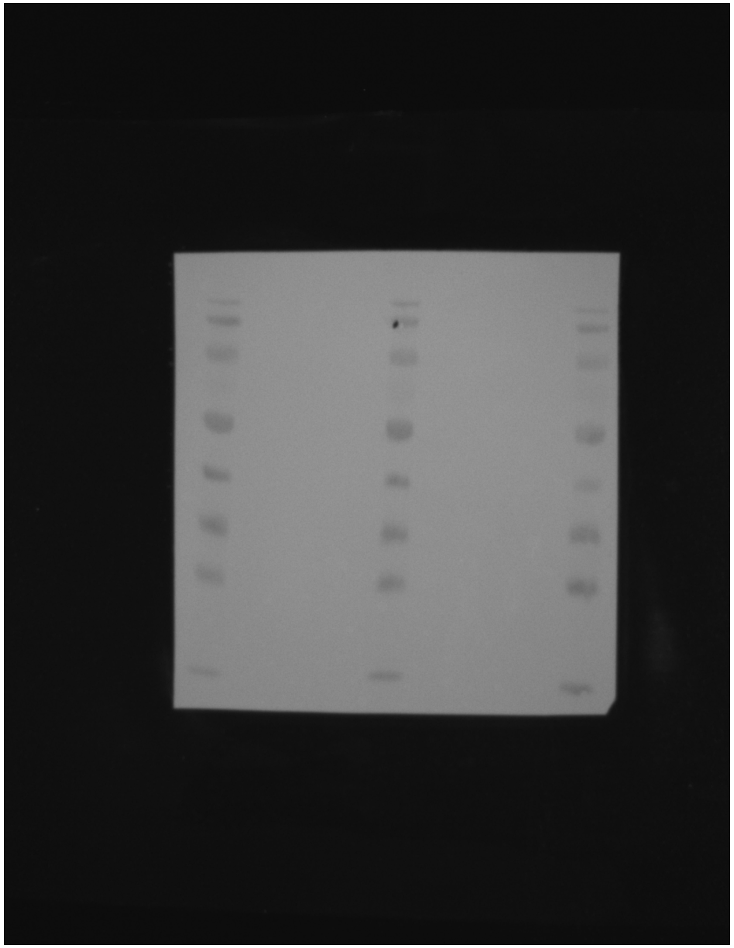

Figure 11c: TNFRSF21 overexpression

Replicate 1: ß-Actin

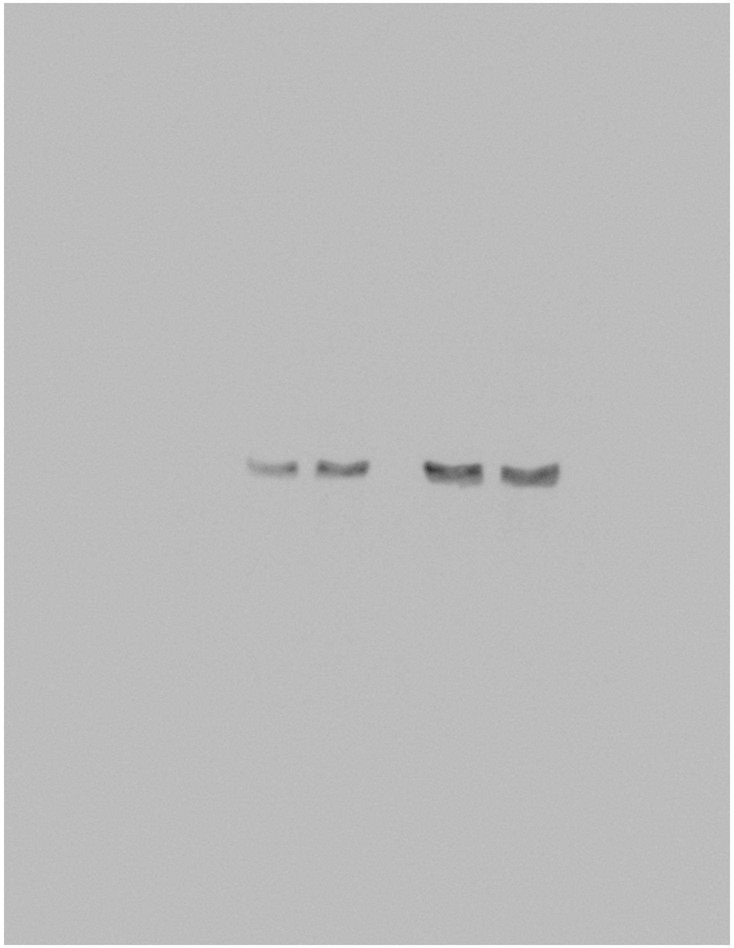

From left to right: empty vector control (30µg protein), TNFRSF21 overexpression (30µg protein), empty vector control (50µg protein), TNFRSF21 overexpression (50µg protein)

Figure 11c: TNFRSF21 overexpression

Replicate 2: marker for TNFRSF21

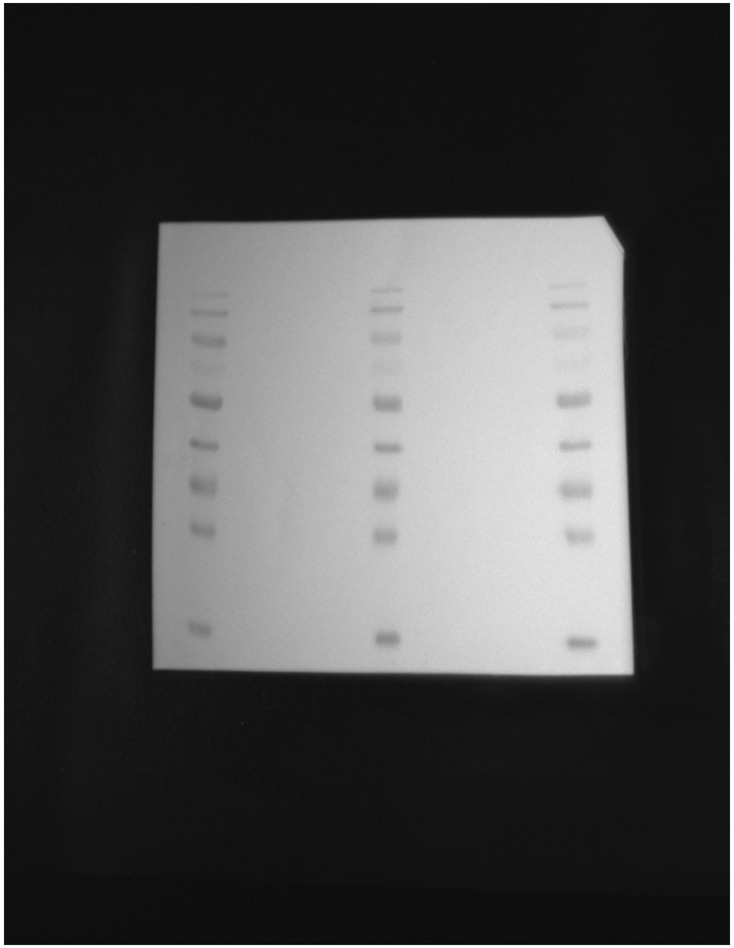

Figure 11c: TNFRSF21 overexpression

Replicate 2: TNFRSF21

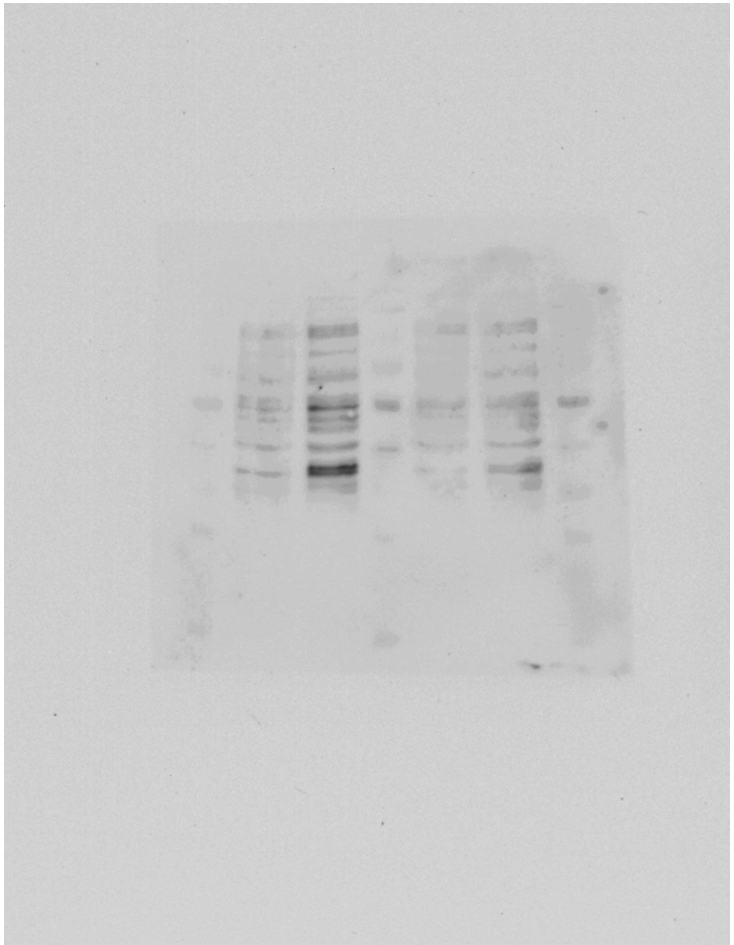

From left to right: empty vector control (replicate 1), TNFRSF21 overexpression (replicate 1), empty vector control (replicate 2), TNFRSF21 overexpression (replicate 2)

Figure 11c: TNFRSF21 overexpression

Replicate 2: marker for ß-Actin

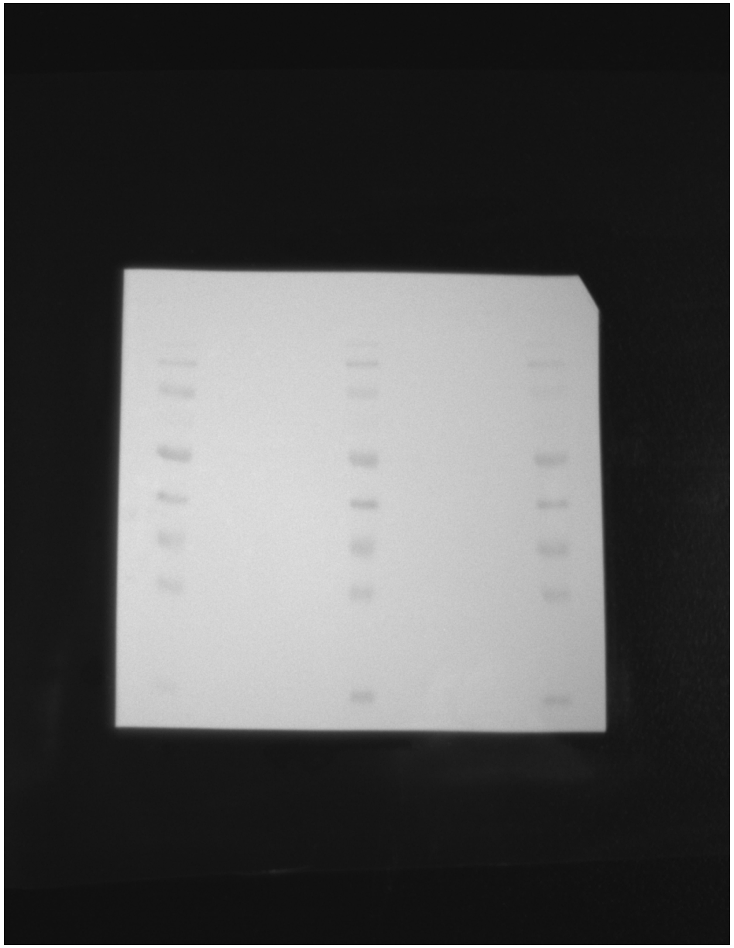

Figure 11c: TNFRSF21 overexpression

Replicate 2: ß-Actin

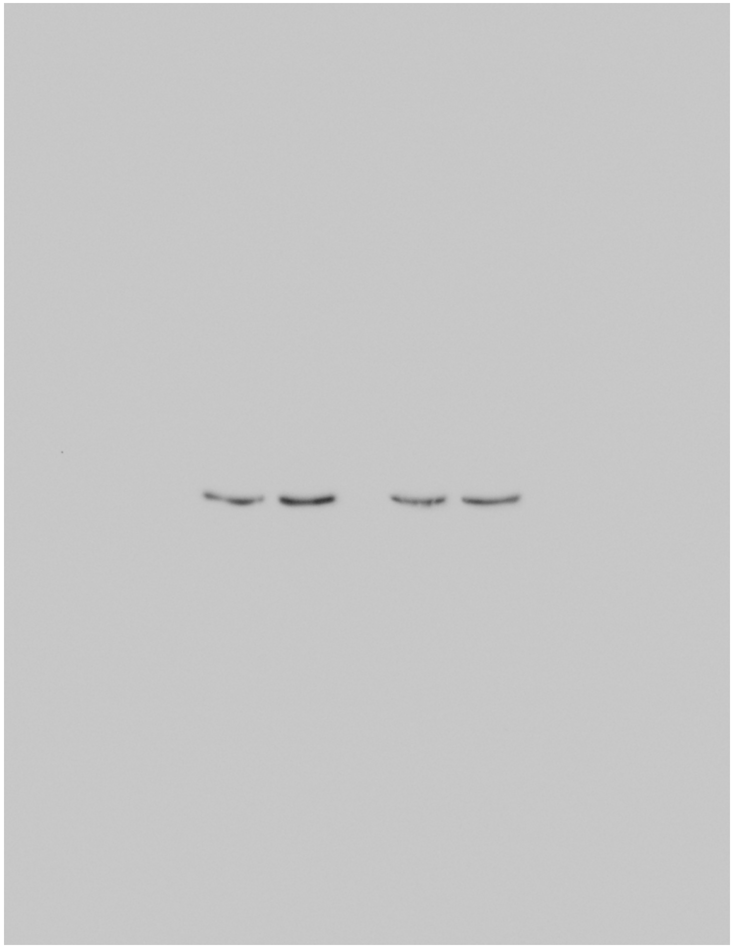

From left to right: empty vector control (replicate 1), TNFRSF21 overexpression (replicate 1), empty vector control (replicate 2), TNFRSF21 overexpression (replicate 2)

Figure 11c: TNFRSF21 overexpression

Replicate 3: marker for TNFRSF21

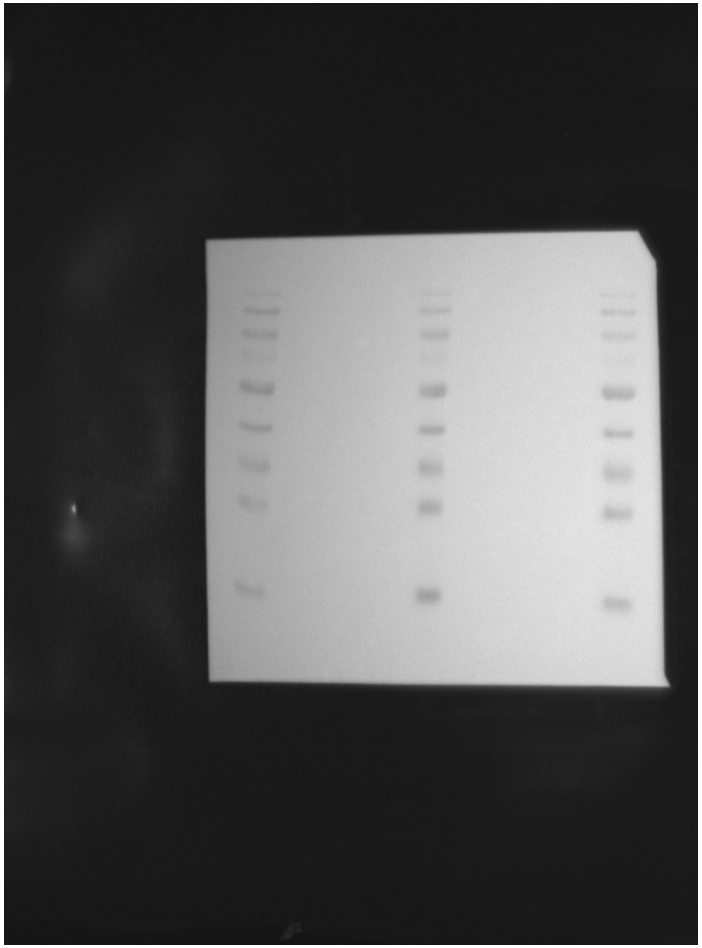

Figure 11c: TNFRSF21 overexpression

Replicate 3: TNFRSF21

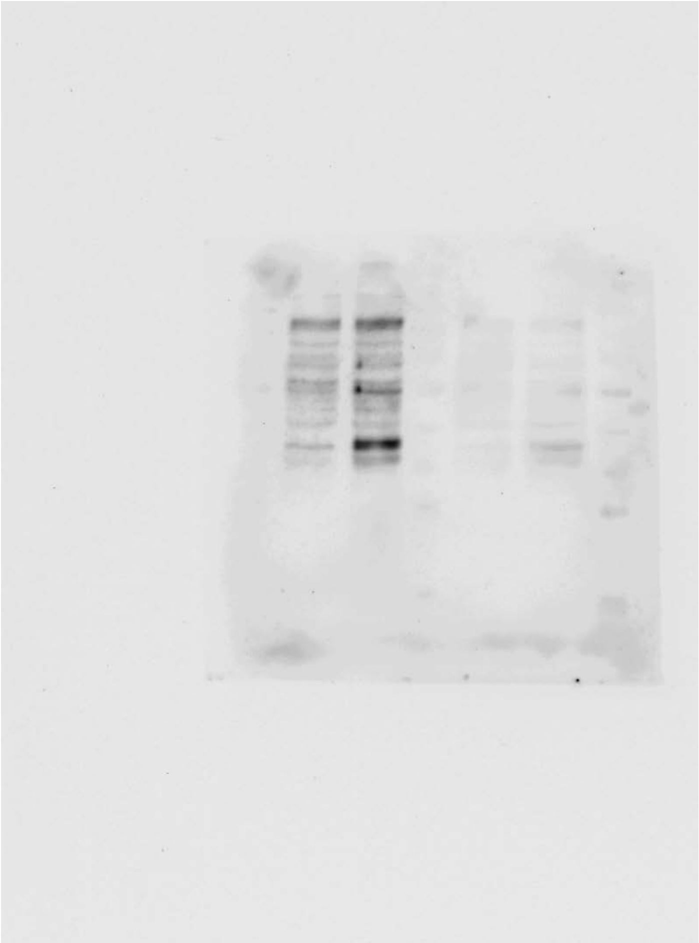

From left to right: empty vector control (replicate 1), TNFRSF21 overexpression (replicate 1), empty vector control (replicate 3), TNFRSF21 overexpression (replicate 3)

Figure 11c: TNFRSF21 overexpression

Replicate 3: marker for ß-Actin

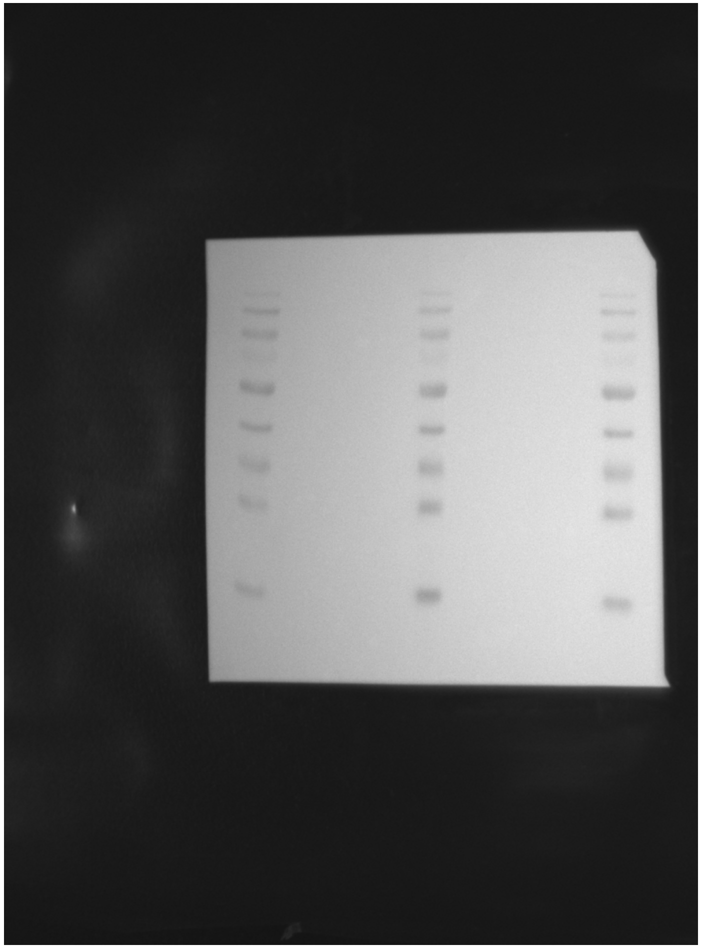

Figure 11c: TNFRSF21 overexpression

Replicate 3: ß-Actin

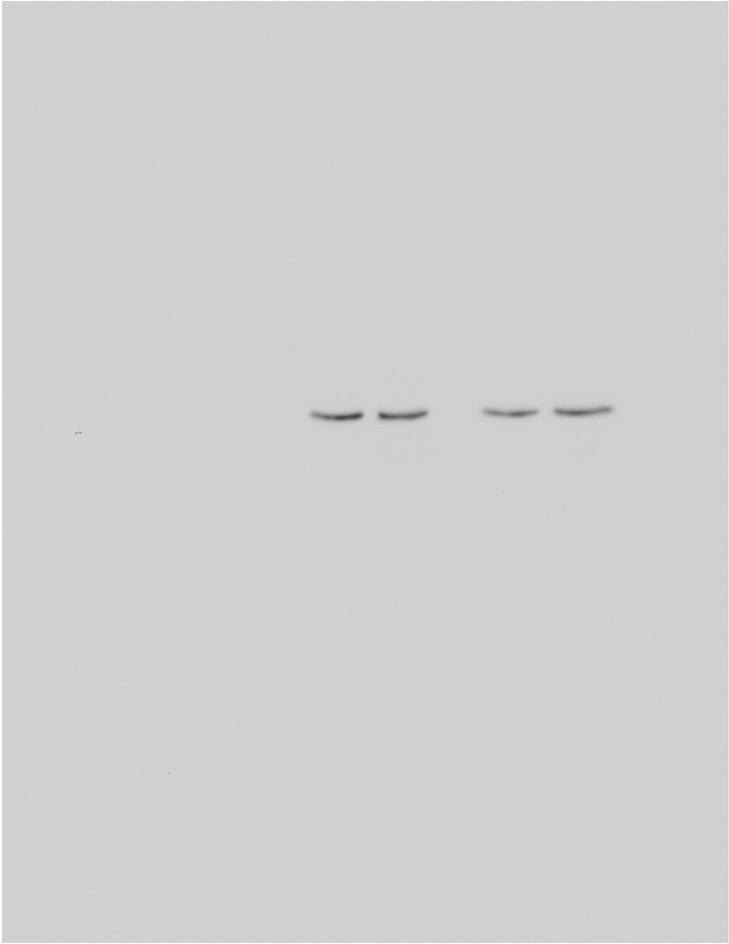

From left to right: empty vector control (replicate 1), TNFRSF21 overexpression (replicate 1), empty vector control (replicate 3), TNFRSF21 overexpression (replicate 3)

Figure 11d: TNFRSF21 endogenous expression

Replicate 1: marker for TNFRSF21

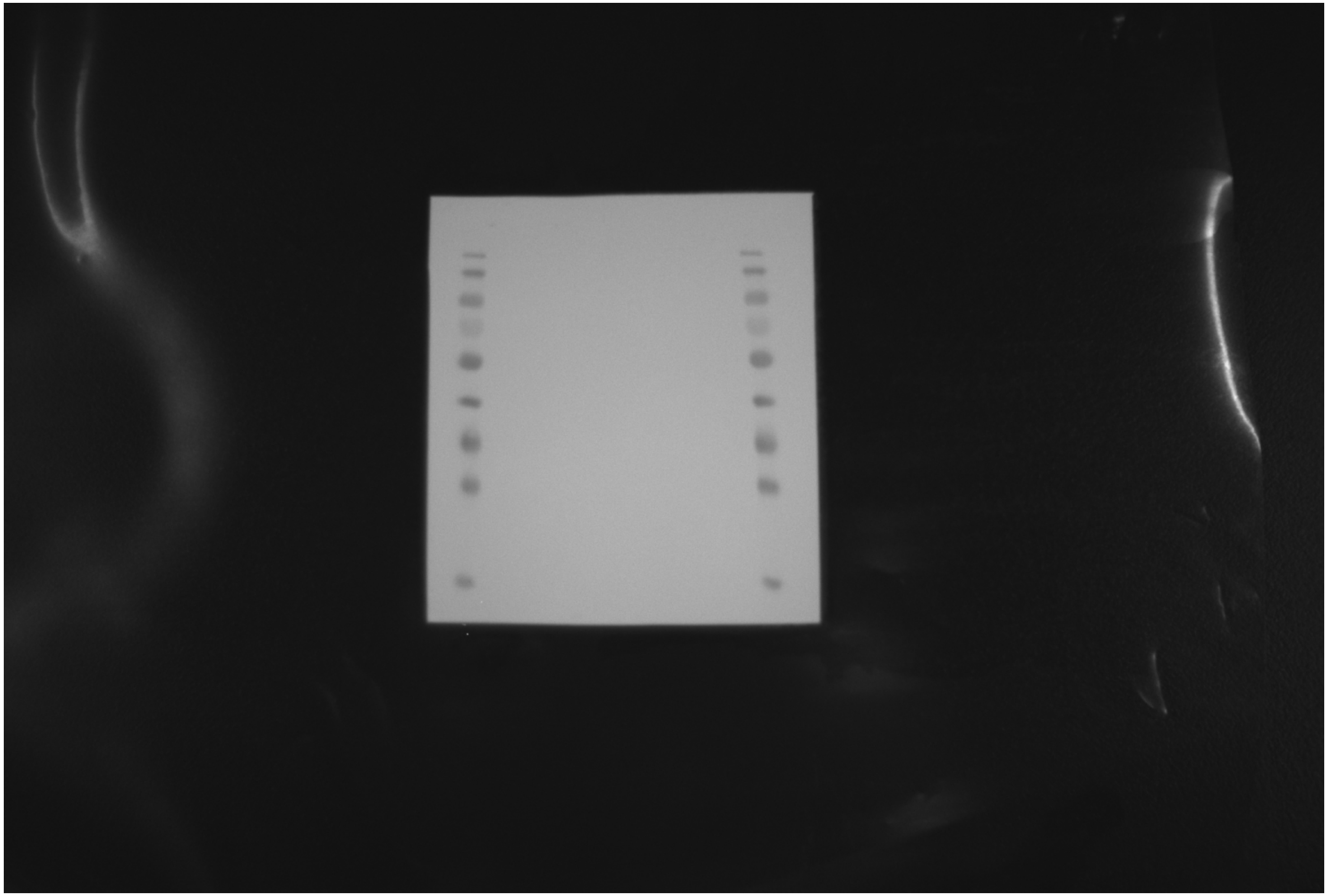

Figure 11d: TNFRSF21 endogenous expression

Replicate 1: TNFRSF21

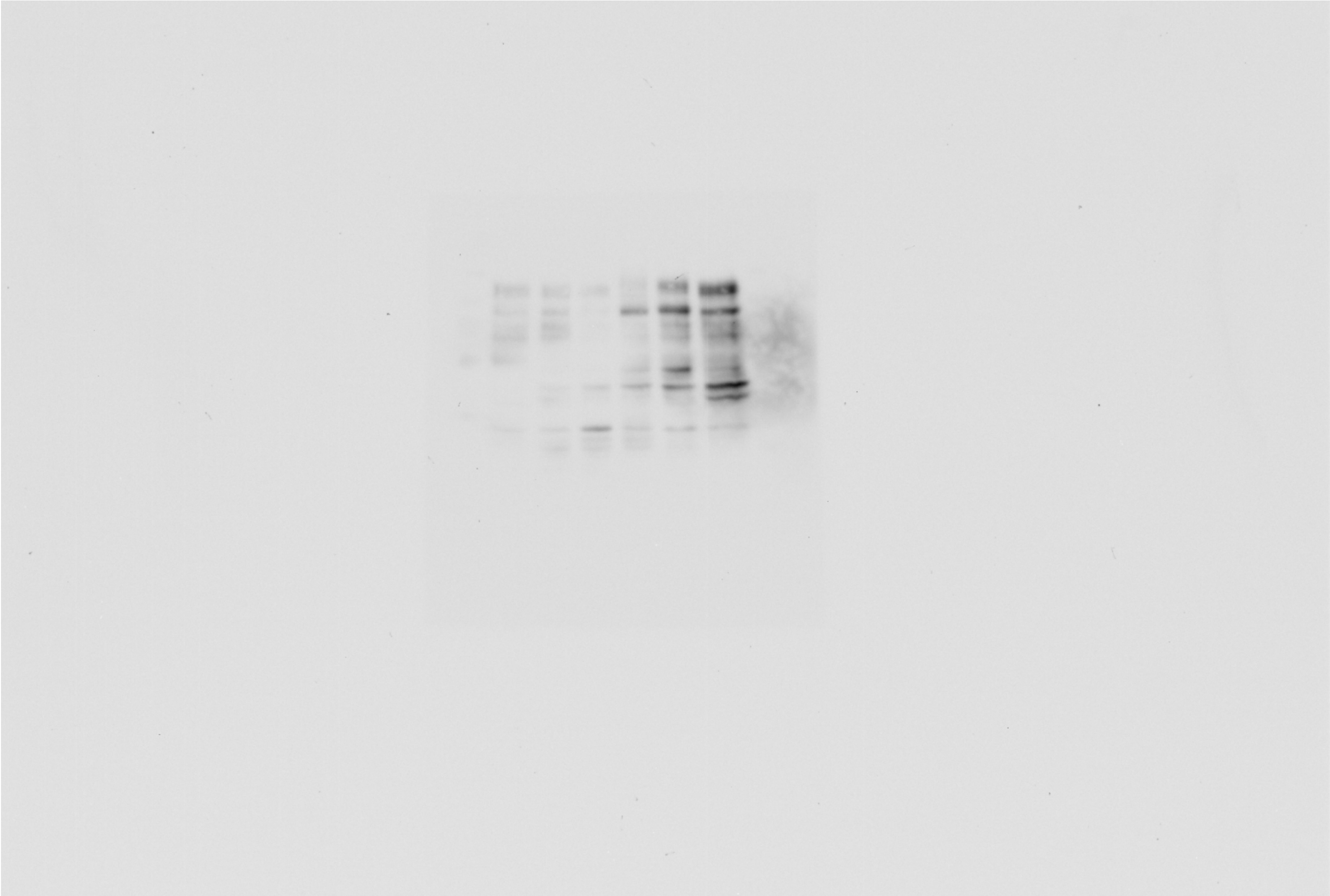

From left to right: NCI-H1792, NCI-H2085, NCI-H1573, NCI-H1648, NCI-H1993, Calu3

Figure 11d: TNFRSF21 endogenous expression

Replicate 1: marker for ß-Actin

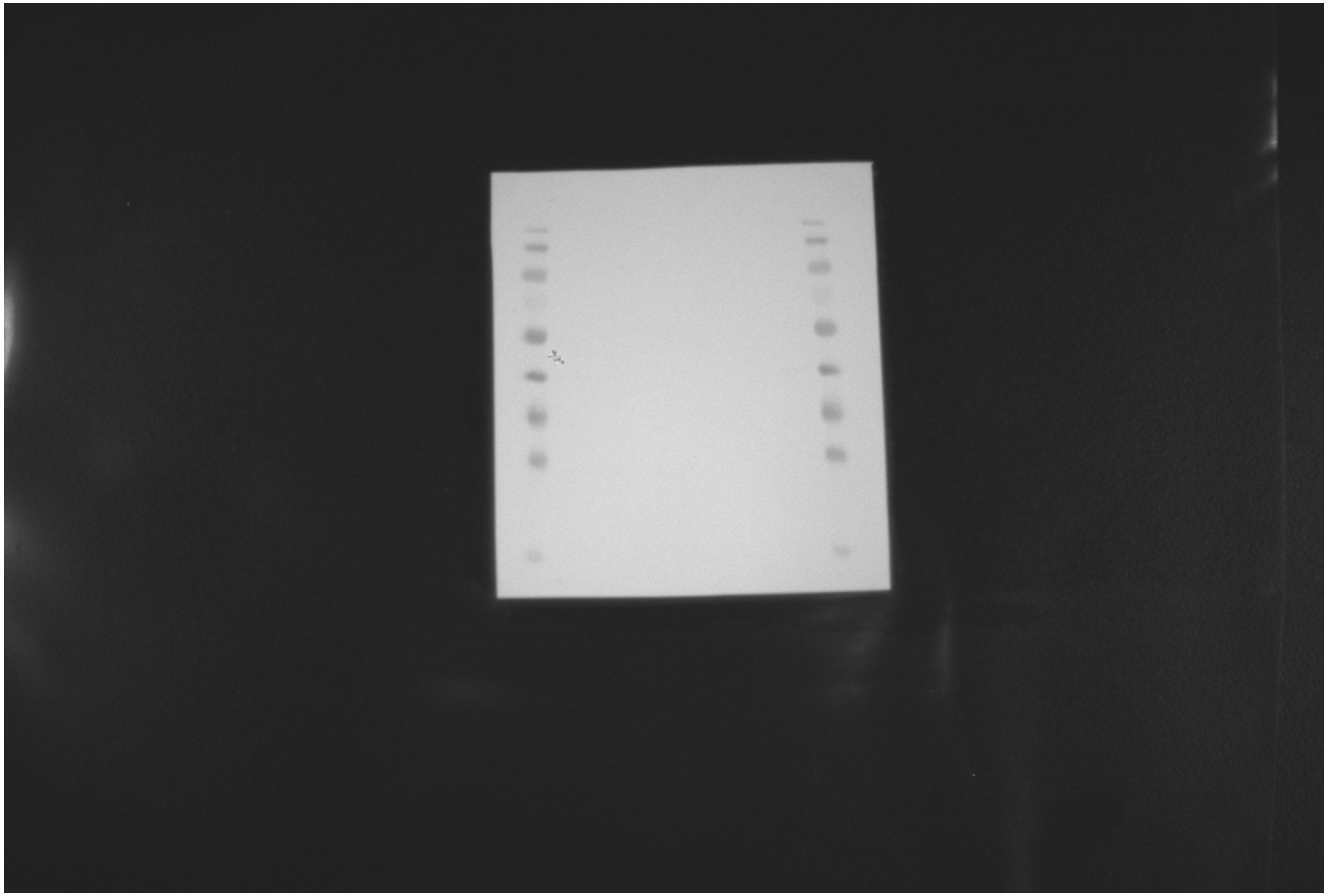

Figure 11d: TNFRSF21 endogenous expression

Replicate 1: ß-Actin

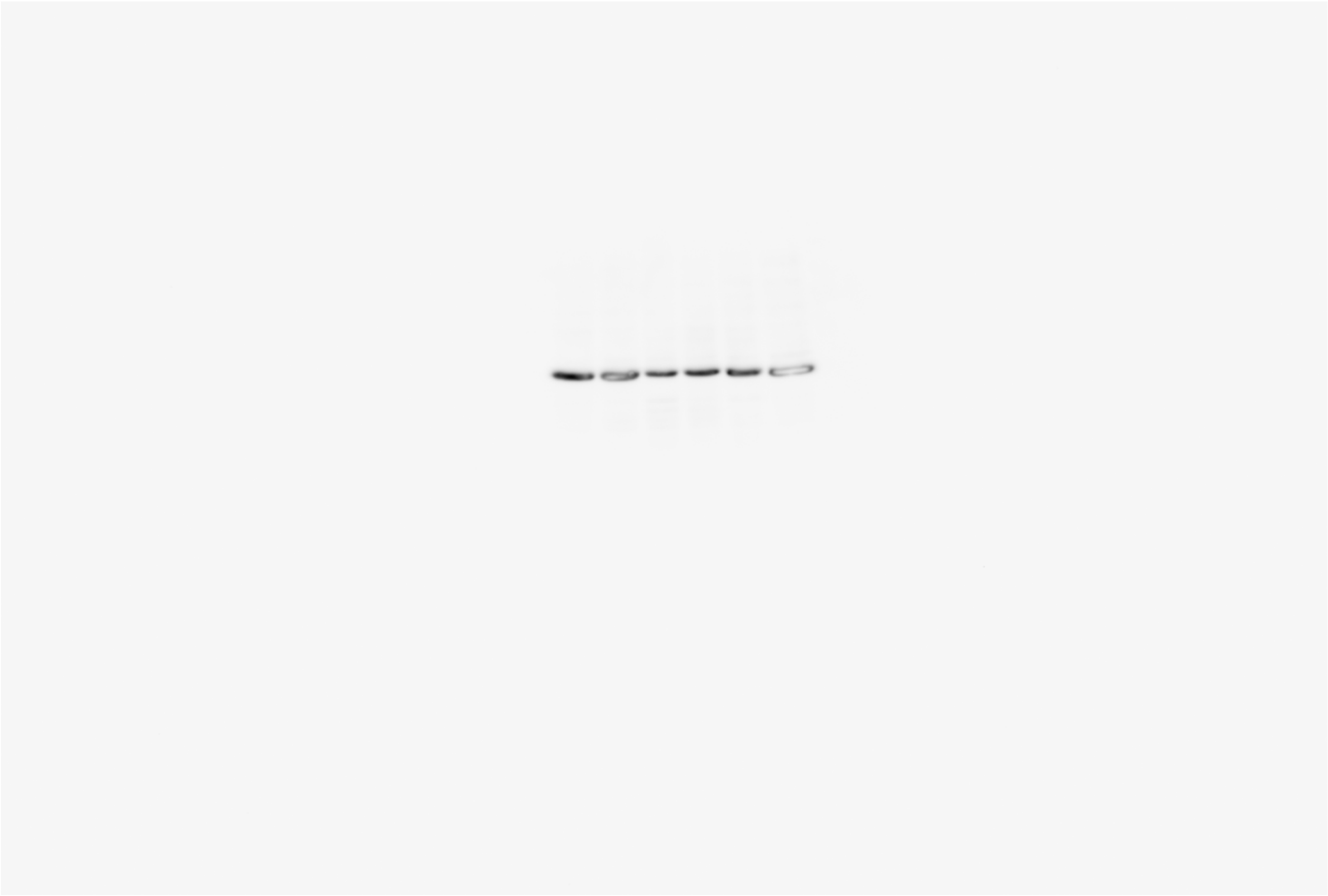

From left to right: NCI-H1792, NCI-H2085, NCI-H1573, NCI-H1648, NCI-H1993, Calu3

Figure 11d: TNFRSF21 endogenous expression

Replicate 2: marker for TNFRSF21

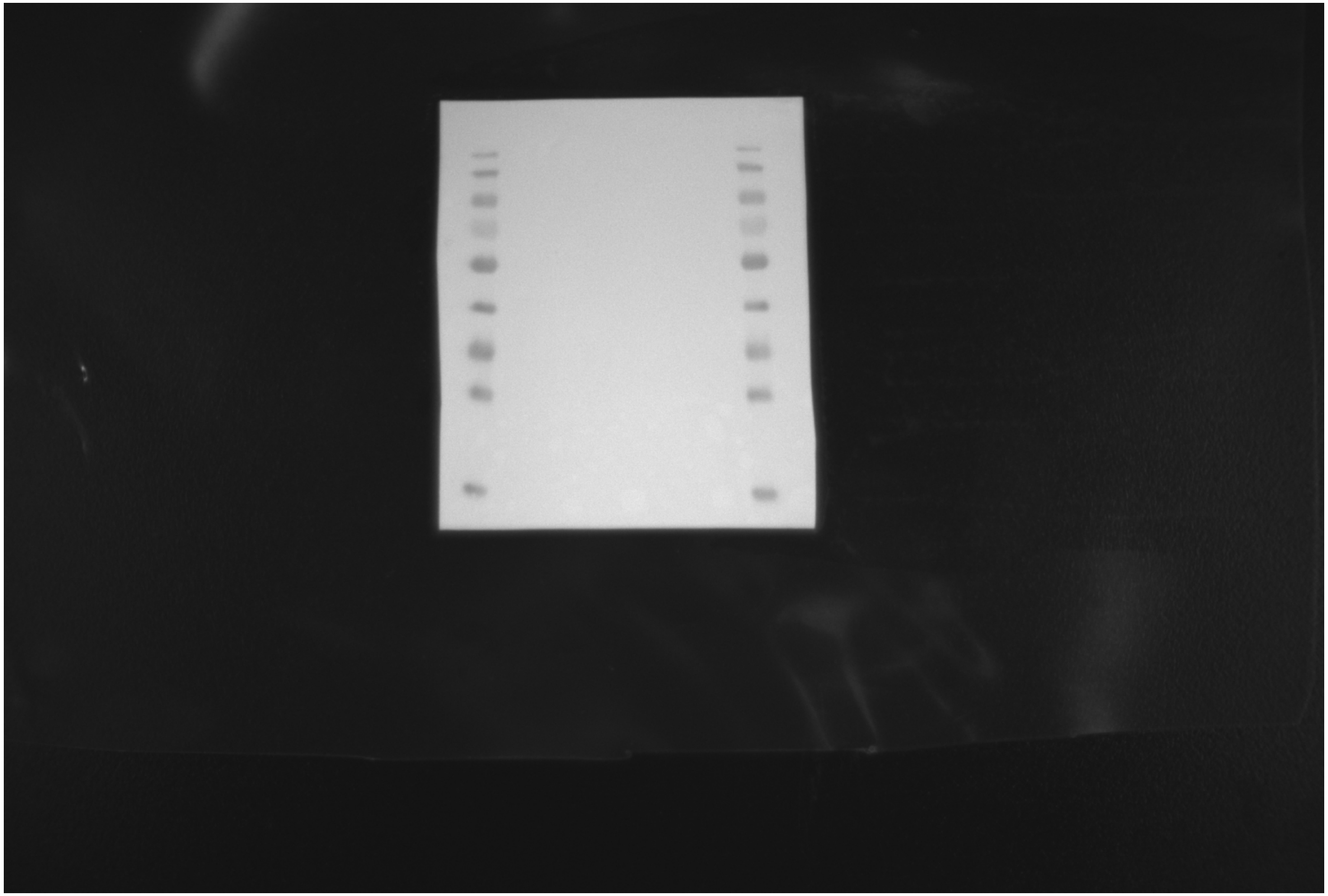

Figure 11d: TNFRSF21 endogenous expression

Replicate 2: TNFRSF21

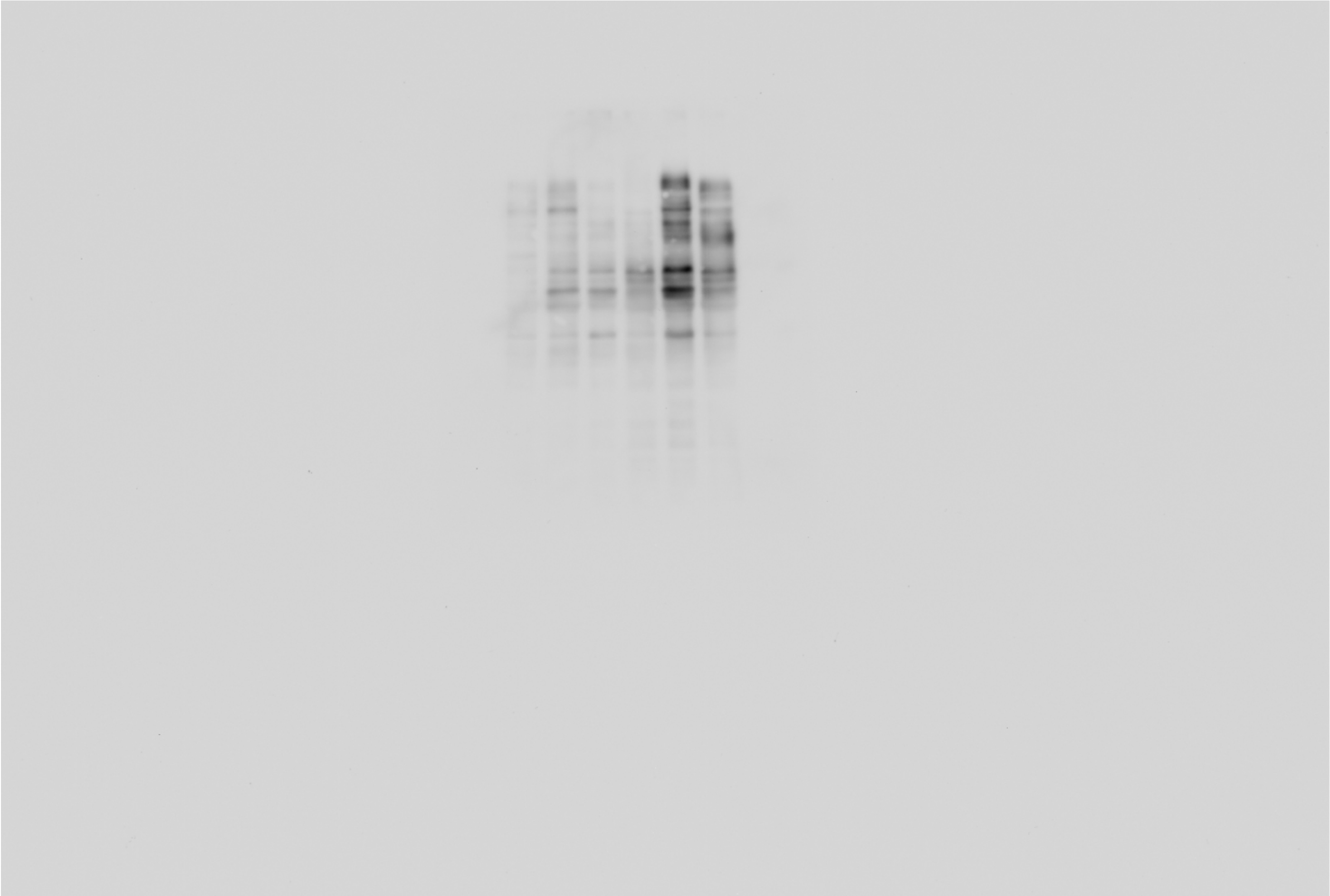

From left to right: NCI-H1792, NCI-H2085, NCI-H1573, NCI-H1648, NCI-H1993, Calu3

Figure 11d: TNFRSF21 endogenous expression

Replicate 2: marker for ß-Actin

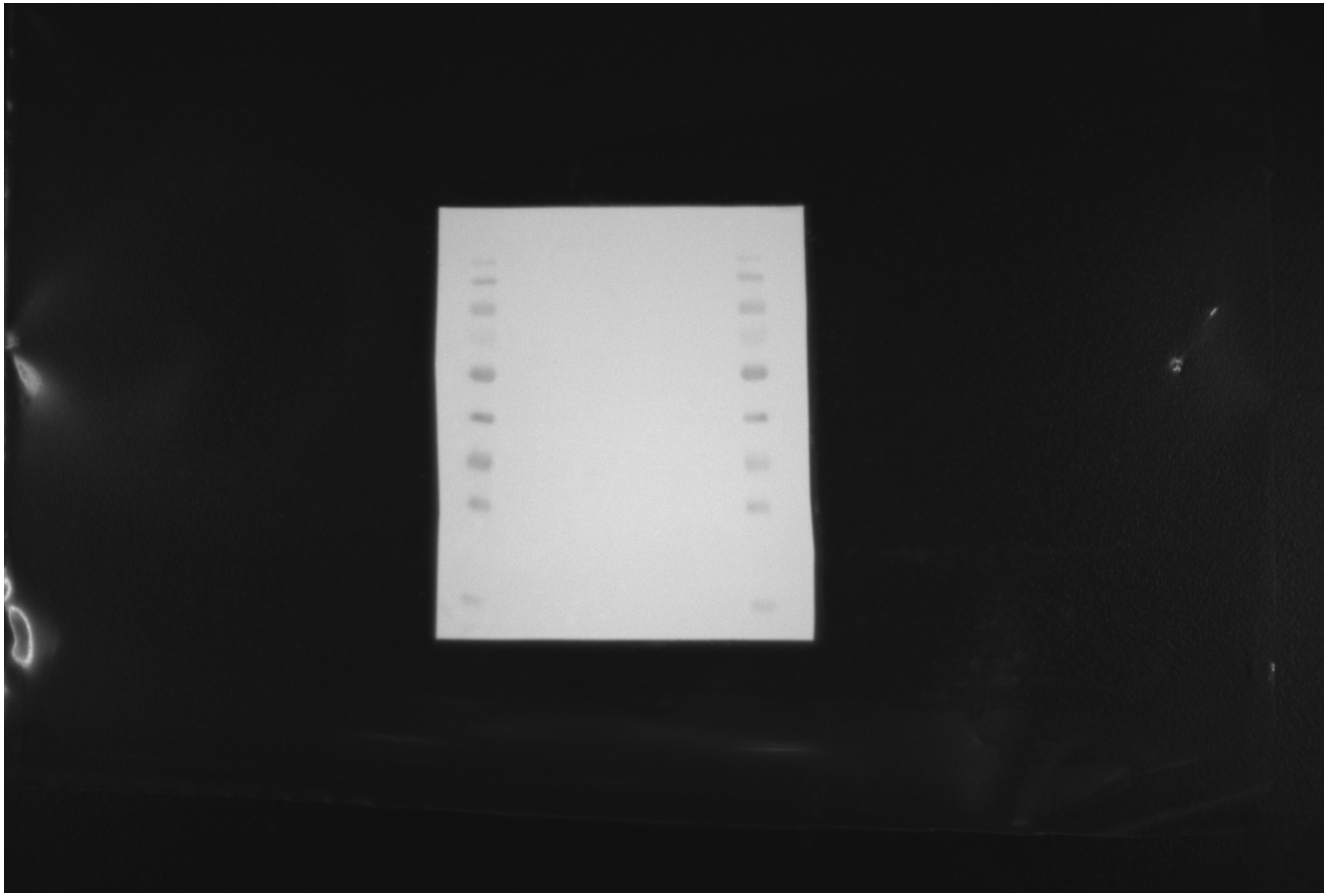

Figure 11d: TNFRSF21 endogenous expression

Replicate 2: ß-Actin

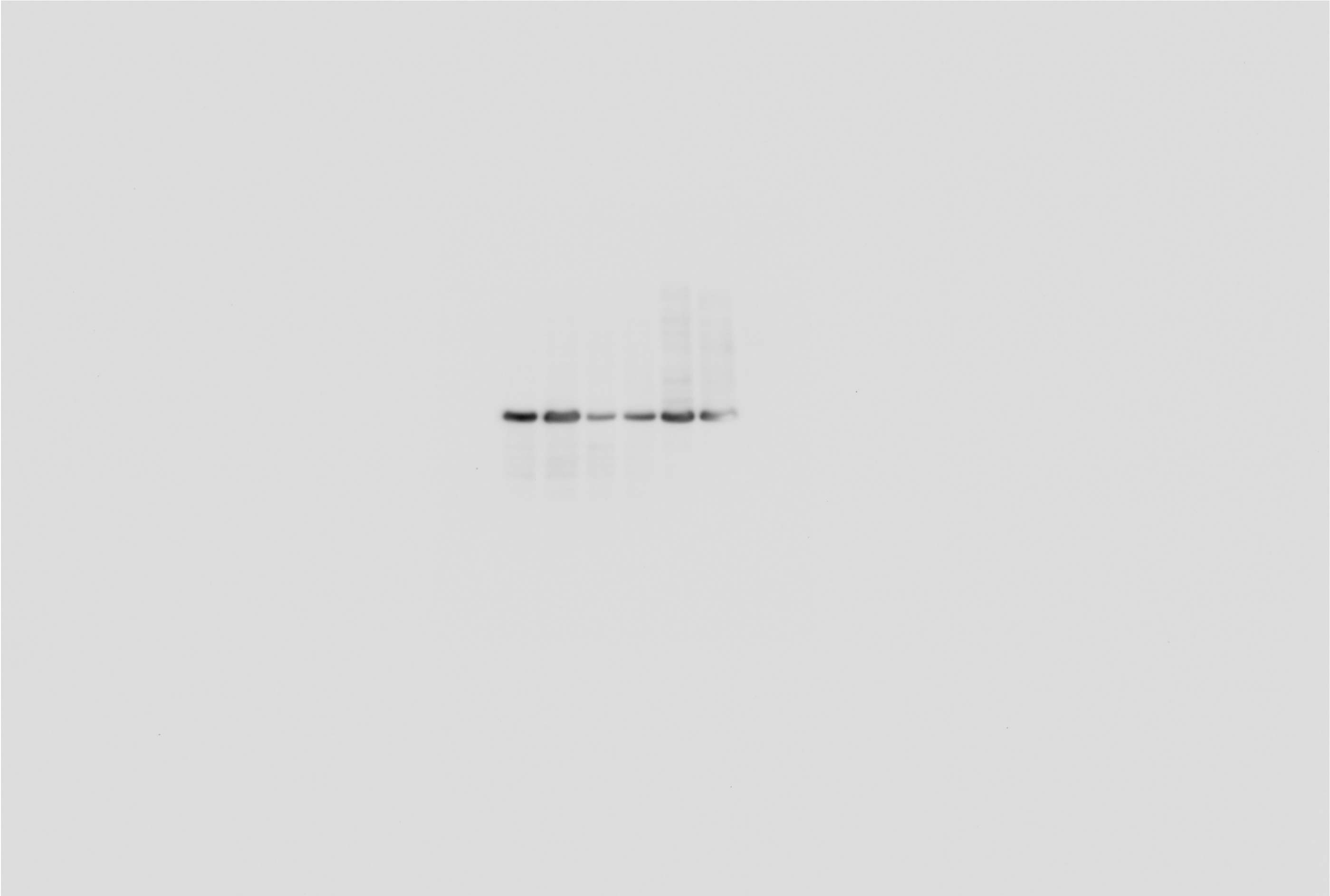

From left to right: NCI-H1792, NCI-H2085, NCI-H1573, NCI-H1648, NCI-H1993, Calu3

Figure 11d: TNFRSF21 endogenous expression

Replicate 3: marker for TNFRSF21

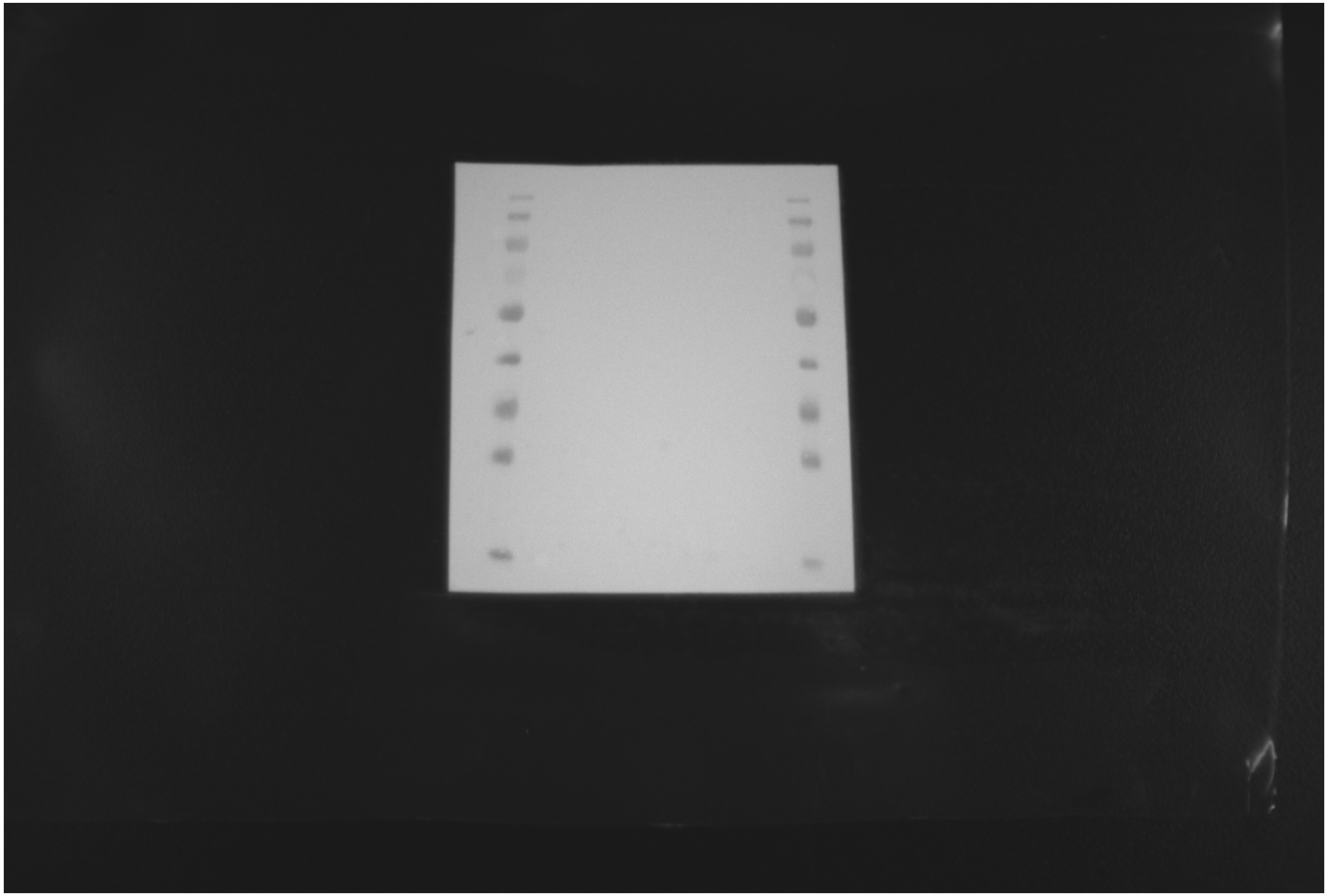

Figure 11d: TNFRSF21 endogenous expression

Replicate 3: TNFRSF21

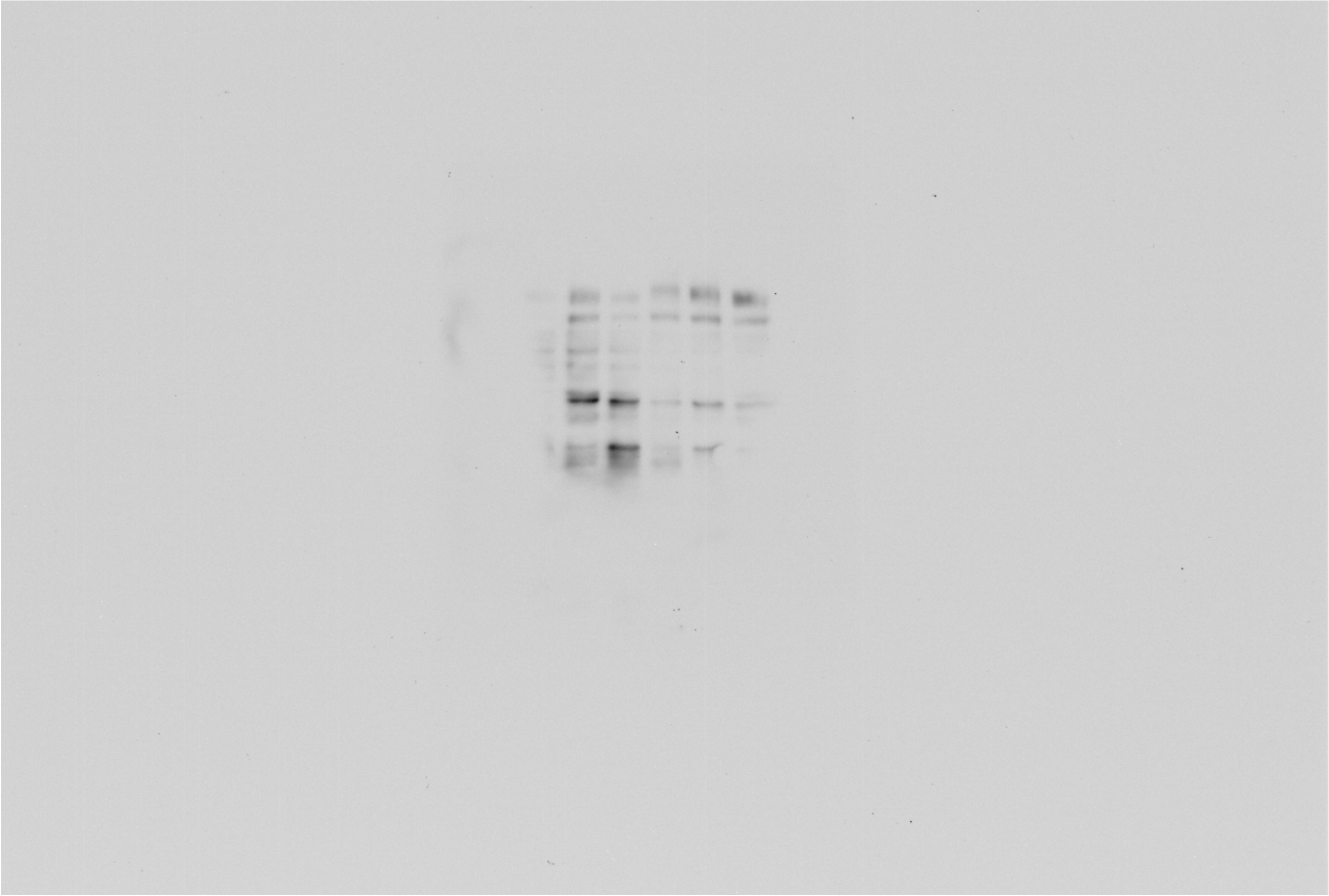

From left to right: NCI-H1792, NCI-H2085, NCI-H1573, NCI-H1648, NCI-H1993, Calu3

Figure 11d: TNFRSF21 endogenous expression

Replicate 3: marker for ß-Actin

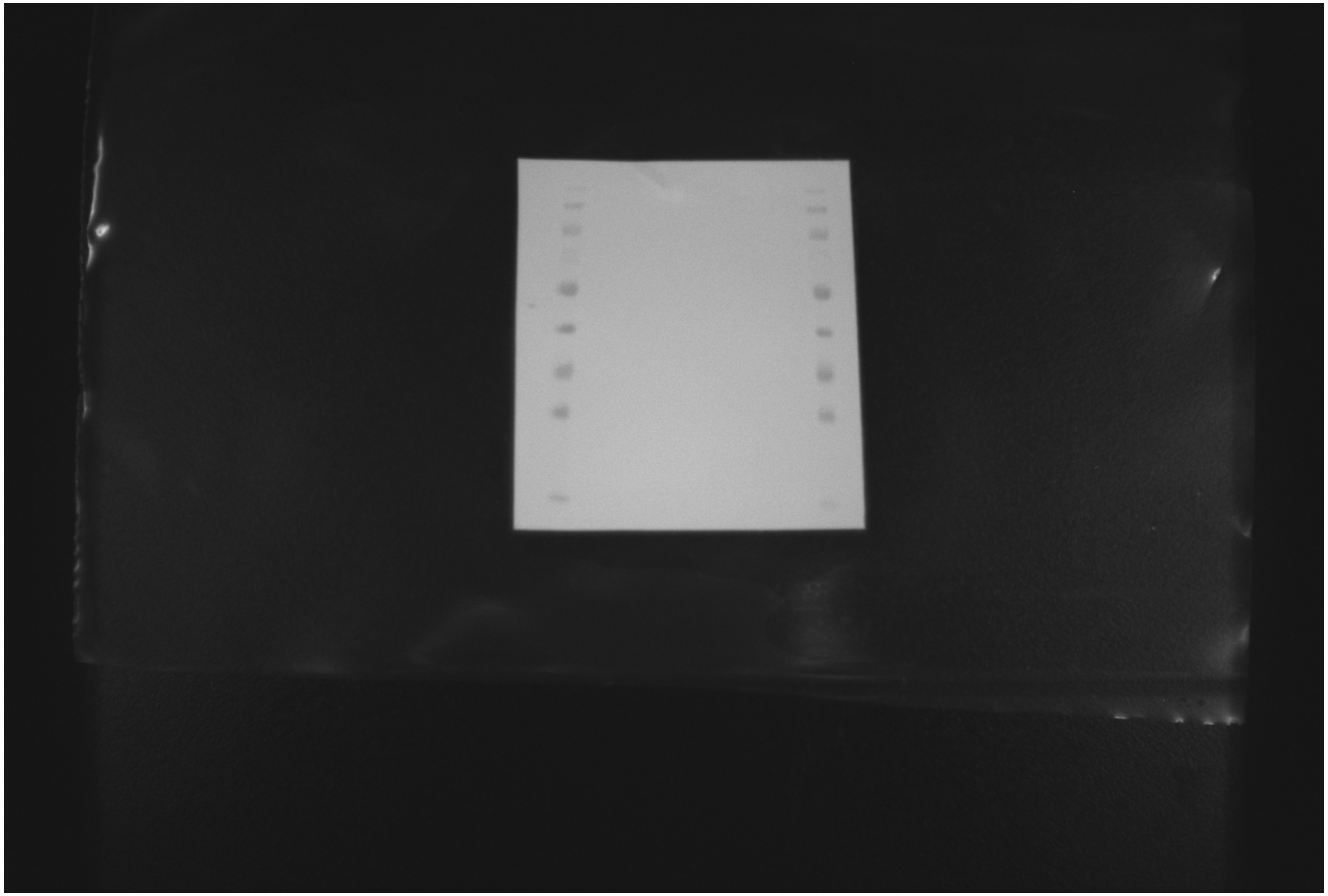

Figure 11d: TNFRSF21 endogenous expression

Replicate 3: ß-Actin

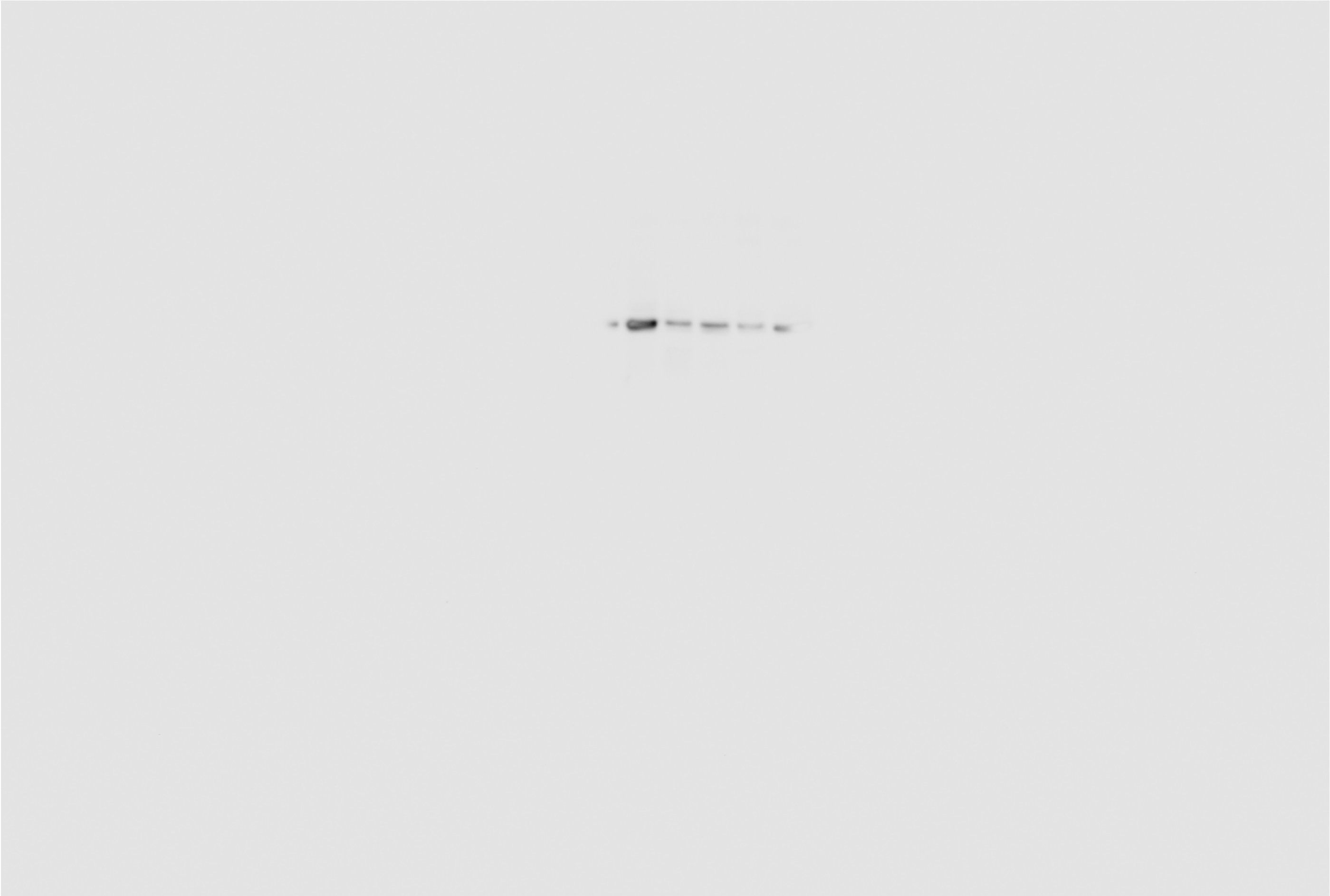

From left to right: NCI-H1792, NCI-H2085, NCI-H1573, NCI-H1648, NCI-H1993, Calu3
